# Fabrication of a poly(*m*‑aminophenol)/3-aminopropyl triethoxysilane/graphene oxide ternary nanocomposite for removal of Cu(II) from aqueous solution

**DOI:** 10.1038/s41598-025-85649-0

**Published:** 2025-01-27

**Authors:** Abeer S. Elsherbiny, Mohamed E. Elhalwagy, Ali H. Gemeay

**Affiliations:** 1 Chemistry Department, Faculty of Science, Tanta University, Tanta, 31527 Egypt; 2 Ethylene Production Sector, Sidi Kerir Petrochemicals Company, Alexandria, Egypt

**Keywords:** Poly (meta-aminophenol), Graphene oxide, Composite, Cu(II) removal, Environmental sciences, Chemistry, Materials science

## Abstract

Three composites based on Poly (meta-aminophenol) (PmAP), (3-aminopropyl) triethoxysilane (APTES) and graphene oxide (GO) were synthesized with initial GO dispersion of 3.3, 6.6, and 9.9 mg/mL. First, in-situ polymerization of meta-aminophenol monomer on the surface of graphene oxide (GO) was carried out. Then, the hydroxyl groups of both the GO and the polymer were targeted using (3-aminopropyl) triethoxysilane (APTES) to stop the polymer solubility, increase adsorption sites, and bind the two components. The obtained three composites were applied for efficient removal of Cu(II) from polluted water. PmAP/APTES/GO_(6.6)_ composite was the best one for the uptake of Cu(II) with a maximum adsorption capacity of 324.54 mg/g at 40 °C and pH 7 according to Langmuir. PmAP/APTES/GO_(6.6)_ hybrid composite was characterized by different techniques. The adsorption of Cu(II) on this composite was optimized under various experimental conditions. Furthermore, the isotherm data of the uptake of Cu(II) on PmAP/APTES/GO_(6.6)_ were found to agree with the Freundlich and Langmuir model’s linear and nonlinear forms. Chemosorption was suggested by the Dubinin-Radushkevich (D-R) isotherm model as the calculated mean sorption energy exceeds 16 kJ/mol. The thermodynamic analysis of the adsorption process reflects an endothermic, spontaneous process that leads to more disorder at the solid-liquid interface. The chemical interactions between Cu(II) versus oxygen and nitrogen of the functional groups on the surface were demonstrated by X-ray Electron Spectroscopy (XPS). Five cycles of adsorption and desorption of Cu(II) from the prepared composite were carried out with a loss of only 6.3% of its adsorption capacity.

**Supplementary Information:**

The online version contains supplementary material available at 10.1038/s41598-025-85649-0.

## Introduction

Potable water is essential for the survival of all living creatures. Water is heavily contaminated and affects human everyday life, agriculture, and industrial growth^1^. Due to the current shortage, water resources have become a worldwide issue, which has caused worry and concern on a global level. Heavy metal water contamination is still a serious environmental problem since it harms ecosystems and human health^2,3^. The quality of residential water will be gravely threatened by high levels of heavy metal ions^4^. The increasing growth of mining, machinery manufacturing, chemicals, electronics, instrumentation, and other sectors produces pollution sources that contain heavy metal ions. Heavy metal ions present in surface water have the potential to cause acute and chronic toxicity on aquatic life since they are difficult to break down into clean, environmentally acceptable molecules^5^. Iron, copper, lead, cadmium, and other excess metal ions induce hypertension, nervous system disorders, and brain illnesses in humans. They also damage bones and kidneys^6–8^. High amounts of Cu(II), which often occur in cationic form, stimulate the digestive tract, resulting in pain, vomiting, and even death^9,10^. Additionally, it has been documented that ingestion of a certain quantity of copper can cause short-term intestinal and stomach issues, liver or kidney damage, and even cancer^11^.

The main producers of copper ions are the mining, textile, battery, and electroplating sectors^12^. According to the United States Environmental Protection Agency (USEPA), industrial effluent should contain no more than 1.3 mg/L of Cu(II) contaminants^13^.

Adsorption^14^, redox reaction^15^, chemical precipitation^16^, coagulation^17^, ion exchange^18^, and membrane filtration^19^ are some methods that have been developed over time for removing heavy metals. The advantages of the adsorption technique over the alternatives are ease of regeneration, cheap cost, and flexibility^20^. Adsorption can also be utilized to reduce and retrieve sources of water for industrial, potable, and other uses. As a result, scientists have continuously worked to develop novel adsorbents that are more effective, quick rate of adsorption, and have great selectivity^21^. Recently, new polymer materials that commonly involve polypyrrole (PPy), polyaniline (PANI), and their derivatives have drawn a lot of interest concerning the removal of dyes, pigments, and different types of pollutants from water. They are useful for adsorbing dyes, heavy metal ions, and other contaminants from sewage and aqueous solutions. In compared to polythiophene (PTh) and polypyrrole (PPy), polyaniline (PANI) and its derivatives have drawn more attention as adsorbents because of their good adsorption capacity, which is owing to the presence of enough amine/imine functional groups in the polymer chains^22^. However, these polymers have certain drawbacks, including weak mechanical strength, a small surface area, few pores, and unstable cycle life. They therefore swell and contract during ion exchange processes, limiting their range of useful uses^23–25^. Poly(m-aminophenol) (PmAP), a substituted derivative of PANI, mostly takes the form of an open ring or ladder-type structure and has two potentially active functional groups (-OH and = NH). These functional groups can help in the removal of dangerous metals from aqueous solutions by chelation^22,26^. When polymer is combined with organic or inorganic components to create a nanocomposite, the adsorption capacity increases and the surface characteristics are improved to be acceptable for the adsorption process^27,28^. Graphene oxide (GO) is a derivative of graphene that has a 2D network assembly. GO has become recognized during the past ten years as a next-generation material for wastewater treatment^29–31^. For the removal of heavy metal ions like Au(III) and Pt(IV)^32^, Pb(II)^33^, Cu(II)^34^, Zn(II)^35^, Cd(II)^36^, and Co(II)^37^, GO is regarded as a promising adsorbent because its corresponding adsorption capacities were significantly higher than those of other adsorbents under similar conditions. However, GO is very dispersible in aqueous media, and employing GO in powdered form might make the aqueous medium hazardous as a result of the leftover GO both before and after the adsorption process. GO is employed as an adsorbent in a variety of ways to address this issue, including three-dimensional GO structures like aerogels^38,39^ and hydrogels^40,41^ and composite GO structures with magnetic nanoparticles^42,43^. Also, using polymers to immobilize GO for example polyaniline/graphene oxide^44^, poly(allylamine hydrochloride) (PAH) with high amine density/graphene oxide^11^, 2,2′-dipyridylamine (DPA)/graphene oxide^45^, graphene oxide/silver phosphate, polyurethane nanocomposite^46^, polyacrylic acid functionalized magnetic iron oxide nanoparticle-graphene oxide nanocomposites^47^, and polypyrrole-cellulose-graphene oxide nanocomposites^48^. Through the grafting-from approach, poly (amidoamine) was utilized to modify GO (GO-PAMAM 2.0), and the modified GO showed outstanding adsorption capacity towards heavy metal ions such Cu(II), Zn(II), Fe(III), Pb(II), and Cr(III)^49^. A further benefit of GO-PAMAM 2.0 over GO in the adsorption of Zn(II), Pb(II), and Cu(II) is the extra complexation of the amines with the heavy metal ions^49^. Highly crystalline Fe_3_O_4_ nanoparticles were deposited onto GO sheets to form Fe_3_O_4_@GO composite which was used for removal of Cu(II) ions^50^. Yao et al. used GO, dialdehyde cellulose and triethylenetetramine to prepare GO-TETA-DAC composite and applied it as a novel adsorbent to remove Cu(II) and Pb(II) ions from aqueous solution. The composite achieved good adsorption ability towards the two ions with maximum adsorption capacity of 65.1 and 80.9 mg/g for Cu(II) and Pb(II), respectively, at pH equals 5^51^. Another modification of GO was done through intercalation GO sheets into bentonite to form bentonite/GO composite. The prepared composite had maximum adsorption capacity of 558.36 and 402.45 mg/g for Cu(II) and Ni(II), respectively based on Langmuir model^52^. Recently, different types of metal-organic frameworks (MOFs) formed composites with GO (MGCs) and were applied to remove heavy metals with greater adsorption efficiency than parent materials^53–60^.

To the best of our knowledge, the modification of PmAP with different amounts of GO has not been reported. Also, the prepared PmAP/APTES/GO_(x)_ ternary nanocomposite has never been utilized for the removal of Cu(II) via the batch adsorption process. Herein, PmAP has been incorporated with GO via an oxidative polymerization process for the removal of Cu(II) ions from water. The solubility of the polymer was eliminated by engaging the free hydroxyl groups in both GO and PmAP in a condensation reaction with the ethoxy group of 3-aminopropyl triethoxysilane (APTES). The physicochemical properties of PmAP/APTES/GO_(6.6)_ ternary nanocomposite were examined using X-ray diffraction (XRD), scanning electron microscopy (SEM), transmission electron microscopy (TEM), thermo-gravimetric analysis (TGA), Fourier transform infrared (FT-IR) spectroscopy, Nitrogen adsorption-desorption measurements, X-ray Electron Spectroscopy (XPS) and Zeta potential techniques. The influence of operation factors such as initial concentration of Cu(II), adsorbent dosage, contact time, pH, and temperature on adsorption efficiency were also determined in this work. In addition, adsorption kinetics and thermodynamics as well as isotherm mechanisms were computed.

## Results and discussion

PmAP could dissolve in water in a greater proportion than 0.2 g/100 mL^61^. The solubility of the polymer was eliminated by engaging the free hydroxyl groups in both GO and PmAP in a condensation reaction with the ethoxy group in APTES. The prepared hybrid was applied for the removal of Cu(II) ions from polluted water. Three different composites were prepared with different constituents of GO. For all of the characterization tests, the hybrid with the highest Cu(II) uptake from the water, PmAP/ APTES/GO_6.6_, in the initial experiments, was chosen.

### Characterization of the composites

#### FT-IR spectra

**Fig. 1:**
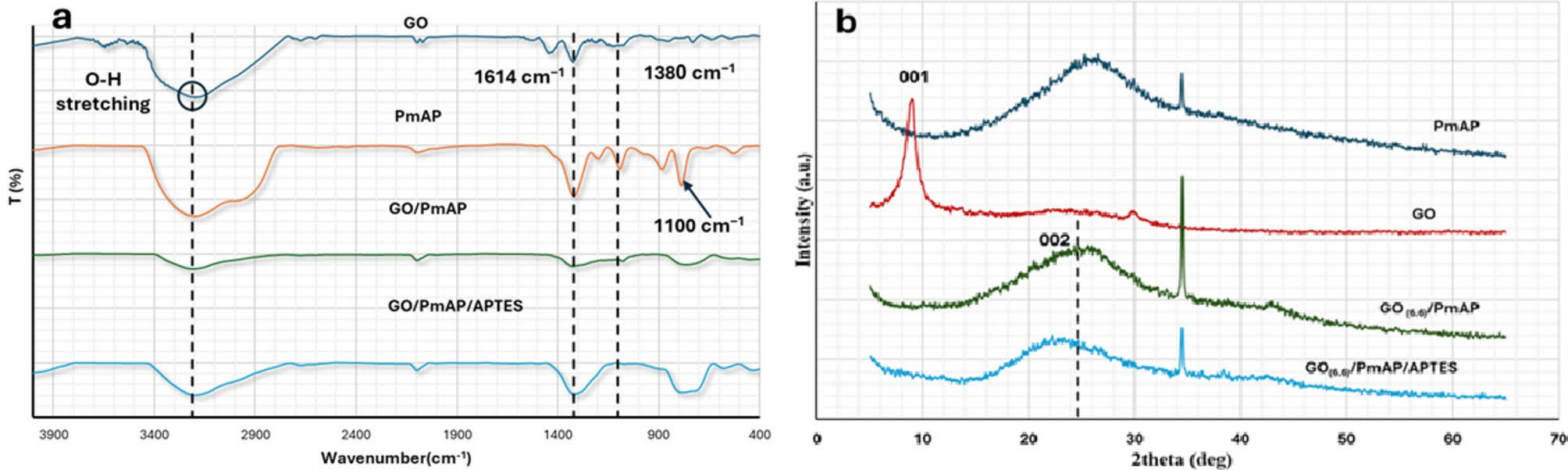
(**a**) FT-IR spectra of GO, PmAP, PmAP/GO_(6.6)_ and PmAP/APTES/GO_(6.6)_; (**b**) XRD for PmAP, GO, PmAP/GO_(6.6)_, and PmAP/APTES/GO_(6.6)_.

FT-IR spectra indicate how the components in the composites are linked to each other and detect the functional groups in the composites. The FT-IR spectra of GO, PmAP, PmAP/GO_(6.6)_ and PmAP/ APTES/GO_6.6_ are shown in Fig. 1a. Considering plain GO data, a broad medium band at 3425 cm^− 1^ is assigned to the O-H stretching vibration. Two weak bands at 2921 and 2851 cm^− 1^ indicate C-H stretching asymmetric and/or symmetric vibration (usually 2–3 bands). The bands at 1821 and 1741 cm^− 1^ refer to C = O stretching vibration connected to aryl or α, β-unsaturated system. The band at 1627 cm^− 1^ is associated with the C = C stretching vibration conjugated with C = O or C = C. An OH deformation vibration band at 1380 cm^− 1^. The band at 1051 cm^− 1^ is due to CO stretching vibration of cyclic diaryl ether of epoxy ring. The broad and diffuse band at 962 cm^− 1^ corresponds to the out-of-plane deformation vibration of carboxylic groups as reported by other authors^62,63^. These all confirm the successful preparation of GO. The FT-IR spectrum of PmAP was quite similar to that published elsewhere^64^. The peak at 3424 cm^− 1^ could be assigned to the OH hydrogen bridge, while the shoulder at 3240 cm^− 1^ corresponds to N-H stretching vibration. The intense band at 1615 cm^− 1^ might be assigned to N-H bending and/or C = N stretching vibration in the polymeric chain (often two bands or a band with a shoulder). The medium and weak bands at 1396 and 1190 cm^− 1^ arise from the C-H deformation vibration of a secondary alcohol. Very little C-O-C linkage was likely produced in the polymers, as predicted by the band at 1100 cm^− 1^^65^. The peaks between 900 and 400 cm^− 1^ correspond to the vibrations of the polymer aromatic rings.

Incorporating PmAP with GO results in a less cluttered spectrum, indicating that the in-situ polymerization of PmAP is adequate for reducing GO^66^. The bands at 3439 cm^− 1^ and a shoulder at 3240 cm^− 1^ are due to the OH hydrogen bridge and the N-H symmetric vibrations, respectively of PmAP. The peak of N-H bending and/or C = N stretching vibration in PmAP appeared at 1614 cm^− 1^. The weak bands at 1378 cm^− 1^ can be assigned C-H deformation vibration of a secondary alcohol. Upon functionalization with APTES, an OH deformation vibration band at 1380 cm^− 1^ was popped out due to the linkage with the ethoxy groups of APTES. As well as all the peaks of the PmAP/GO_(x)_ composite appeared to demonstrate that APTES grafting on PmAP/GO_(x)_ contributes to PmAP stabilization.

#### XRD patterns

XRD patterns distinguish between the crystalline and amorphous shapes of solids as well as the change in the planes of the crystalline materials upon external factors. Figure 1b presents the XRD of PmAP, GO, PmAP/GO_(6.6)_, and PmAP/APTES/GO_(6.6)_. On the XRD of the PmAP polymer, crystalline and amorphous diffraction patterns were observed^67^. A relatively sharp peak at ($\:2{\uptheta\:}=34.53^\circ\:$) indicates the presence of crystalline regions as a result of the presence of amine and phenol groups. These groups form strong hydrogen bonds and electrostatic dipole interactions between the chains^68^. A wide peak between two values of 20–30°, centered at $\:2{\uptheta\:}$ = 25.2°, can be attributed to an open ring planar structure comprising benzenoid and quinoid units.

The bare GO exhibits a typical peak at $\:2{\uptheta\:}=9.12^\circ\:$ corresponding to the plane (001)^69^. After the PmAP polymerization was completed in the presence of GO, the sharp peak of PmAP at$\:\:2{\uptheta\:}=34.53^\circ\:$ had appeared. However, the (001) peak of GO vanished and a new peak was noticed at $\:2{\uptheta\:}=25.22^\circ\:$ corresponding to the (002) plane which is characteristic of reduced GO (rGO)^70^. This suggests that basic medium and amine groups have caused GO sheets to be chemically reduced^71^. This peak has fused with its counterpart in the polymer. These all confirm the polymerization of PmAP on the GO surface. As a consequence of functionalizing PmAP/GO_(6.6)_ with APTES, the peak at $\:2{\uptheta\:}=25.22^\circ\:$ (d_002_ = 7.06 Å) was shifted to 23.18$\:^\circ\:$ (d_002_ = 7.35 Å) revealing the intercalation of PmAP/APTES between rGO layers. Again, the polymer’s characteristic crystalline peak at $\:2{\uptheta\:}=34.53^\circ\:$ appeared.

#### N_2_ adsorption-desorption measurement

**Fig. 2:**
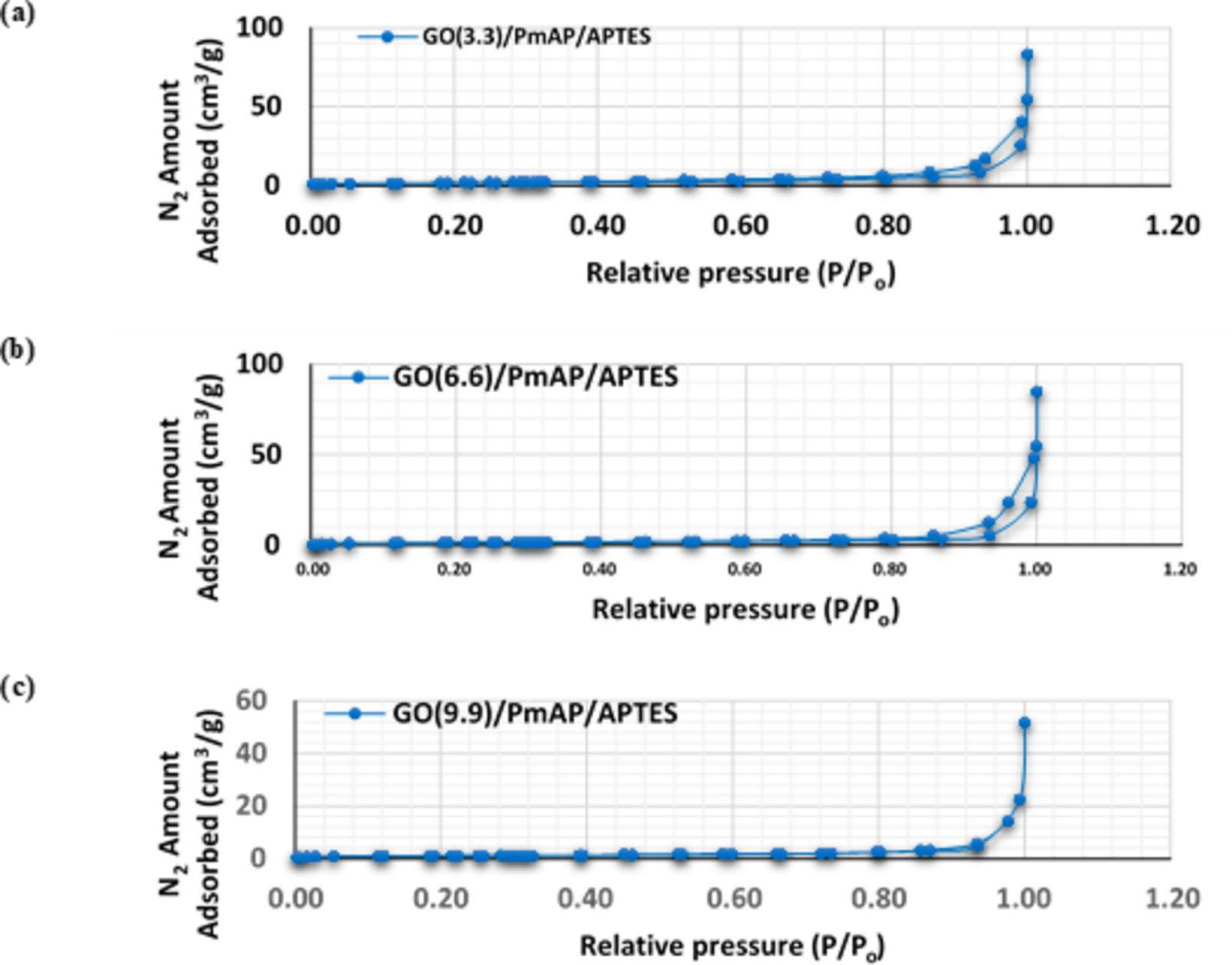
N_2_ adsorption-desorption isotherm of (**a**) PmAP/APTES/GO_(3.3)_, (**b**) PmAP/APTES/GO_(6.6)_ ; (**c**) PmAP/APTES/GO_(9.9)_.

N_2_ adsorption-desorption measurement is an important technique to determine the surface area, pore size and pore volume of solid materials. As well as the type of materials according to their pore size. Brunauer-Emmett-Teller (BET) surface area analysis and Barrett-Joyner-Halenda (BJH) pore size and volume analysis were carried out for the final samples $\:\text{P}\text{m}\text{A}\text{P}/\text{A}\text{P}\text{T}\text{E}\text{S}/{\text{G}\text{O}}_{\left(\text{x}\right)}\:$originated with different GO dispersion (x). The hybrid’s surface area gradually increases as the proportion of GO increases to a certain extent. As the proportion of GO increased in the $\:\text{P}\text{m}\text{A}\text{P}/\text{A}\text{P}\text{T}\text{E}\text{S}{/\text{G}\text{O}}_{\left(3.3\right)}$ and $\:\text{P}\text{m}\text{A}\text{P}/\text{A}\text{P}\text{T}\text{E}\text{S}/{\text{G}\text{O}}_{\left(6.6\right)}$, the recorded surface area was 60.85 and 68.55 m^2^/g, respectively. Conversely, with the further increase in the $\:\text{P}\text{m}\text{A}\text{P}/\text{A}\text{P}\text{T}\text{E}\text{S}{/\text{G}\text{O}}_{\left(9.9\right)}$ sample, the surface area dramatically decreased to 36.89 m^2^/g. Considering the BJH, it was observed that the average pore size also increased from 34.94 to 63.26 nm as the initial concentration of the GO increased from 3.3 to 6.6 mg/L. However, it regressed in the final sample $\:\text{P}\text{m}\text{A}\text{P}/\text{A}\text{P}\text{T}\text{E}\text{S}{/\text{G}\text{O}}_{\left(9.9\right)\:}$ to 21.64 nm as GO became the main constituent. Regarding the study of the N_2_ desorption-adsorption isotherm, samples $\:\text{P}\text{m}\text{A}\text{P}/\text{A}\text{P}\text{T}\text{E}\text{S}{/\text{G}\text{O}}_{\left(3.3\right)}$ and$\:\:\text{P}\text{m}\text{A}\text{P}/\text{A}\text{P}\text{T}\text{E}\text{S}{/\text{G}\text{O}}_{\left(6.6\right)}$ exhibit a typical mesoporous type (V) isotherm (IUPAC classification), Fig. 2(a, b). While the final sample $\:\text{P}\text{m}\text{A}\text{P}/\text{A}\text{P}\text{T}\text{E}\text{S}{/\text{G}\text{O}}_{\left(9.9\right)}$tends to be of the macroporous or nonporous type (III)^72^, Fig. 2(c). There is also a relationship between the hysteresis loop’s form and the distribution of pore size, shape, and interconnectivity. IUPAC proposed a taxonomy of empirical hysteresis loops H(1–4). For $\:\text{P}\text{m}\text{A}\text{P}/\text{A}\text{P}\text{T}\text{E}\text{S}{/\text{G}\text{O}}_{\left(3.3\right)}$and $\:\text{P}\text{m}\text{A}\text{P}/\text{A}\text{P}\text{T}\text{E}\text{S}{/\text{G}\text{O}}_{\left(6.6\right)}$ samples are the following H3 hysteresis materials. Such materials have holes in the shape of slits, which can be seen in groups of plate-like particles that are not rigid^72^. Mesoporous graphene-based materials have previously been demonstrated^73^.

#### Thermal analysis

The thermal stability of the prepared materials can be determined using thermal gravimetric analysis (TGA). The new hybrid $\:\text{P}\text{m}\text{A}\text{P}/\text{A}\text{P}\text{T}\text{E}\text{S}{/\text{G}\text{O}}_{\left(6.6\right)}$, in addition to the intermediate composites, were subjected to Thermal gravimetric and Derivative Thermal gravimetric (DTG) analysis. TGA measurements were acquired for all samples under conditions of a 10 °C min^− 1^ heating rate, a temperature range up to 800 °C, and a 10 mL min^− 1^ nitrogen flow rate. The TGA curve of GO can be broken down into three distinct phases^69^, Fig. S1. The degassing and evaporation of the adsorbed moisture account for the initial weight loss of 21.41% up to 150 °C. The decomposition of the mutable oxygen-containing groups causes a subsequent significant weight loss of 67.32% between 150 and 370 °C. However, the weight loss of 7.03% between 370 and 650 °C was assigned to the decomposition of relatively stable oxygen-containing groups. GO lost about 89.83% of its original weight after reaching 800 °C.

Figure S2 shows the typical TGA curve of the PmAP^68^. The sample expelled both adsorbed gases and moisture at temperatures up to 120 °C. Following this step, a weight loss of 10.7% between 120 and 244 °C was observed, corresponding to the sublimation of the oligomer from the polymer matrix. A third mass loss of 14.43% occurs between 244 and 403 °C, corresponding to the exit of the dopants from the polymer matrix^74^. After 403 °C, the polymer side structures started to break down. At 658 °C, the rate of degradation begins to accelerate, indicating the decomposition of the main polymer chain structure. At 800 °C, 42.7% of the polymer’s weight still existed.

Figure S3 depicts the TGA-DTA curve for $\:\text{P}\text{m}\text{A}\text{P}/{\text{G}\text{O}}_{\left(6.6\right)}\:$after in situ polymerization of mAP within GO. The curve displays a thermal decomposition that is closer to polymer than to GO. GO might be converted into reduced GO due to the alkaline polymerization conditions as well as the presence of an amine^71^. This might result in the fading of the oxygen-containing groups’ degradation zones^75^. Consequently, the remaining weight of 45.42% was comparable to the gravimetric calculation of 42.51% for the GO constituents. In addition, the degraded portion of 54.58% was comparable to the polymer constituents of 57.49%.

**Fig. 3:**
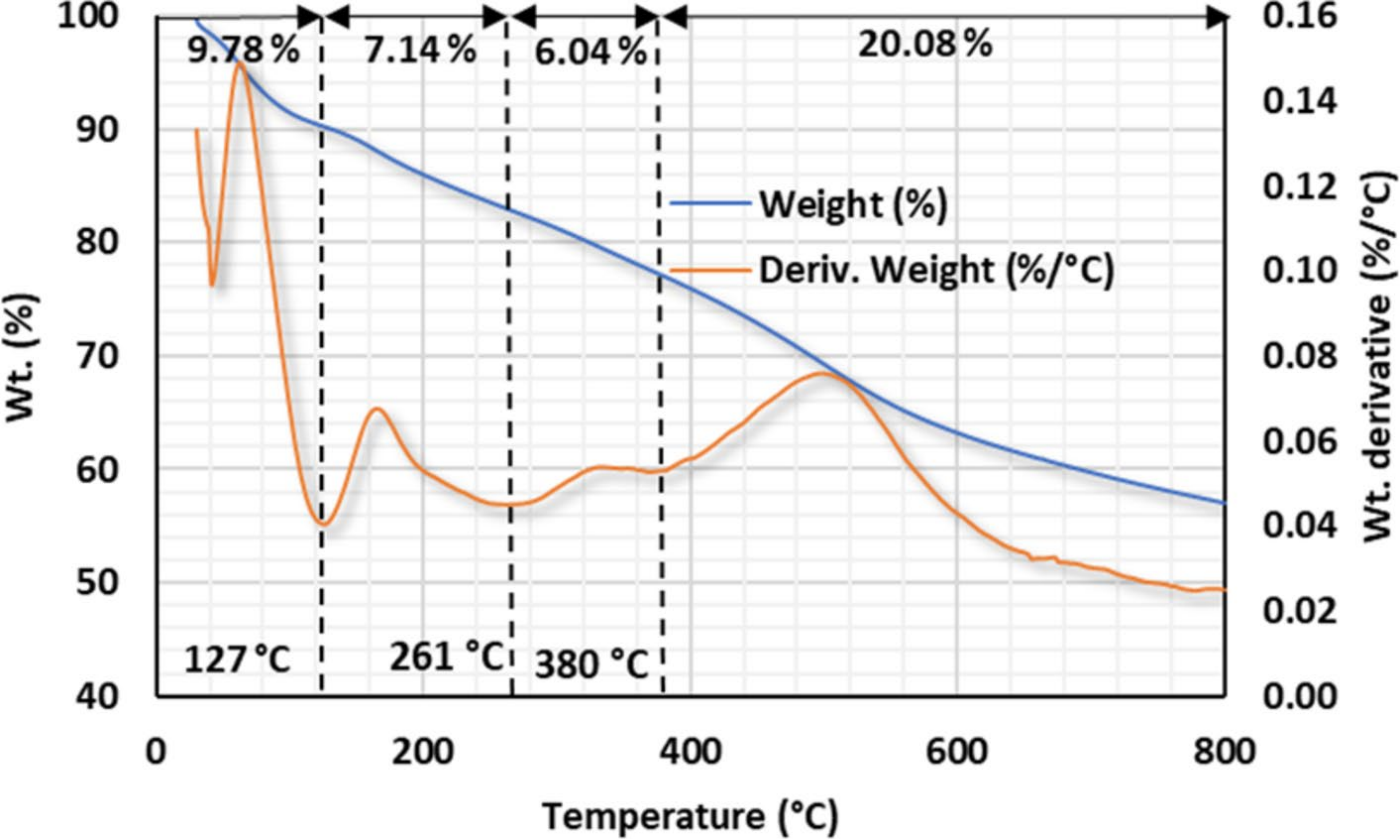
TGA-DTA curve for $\:\text{P}\text{m}\text{A}\text{P}/\text{A}\text{P}\text{T}\text{E}\text{S}{/\text{G}\text{O}}_{\left(6.6\right)}$.

Figure 3 presents the TGA-DTA spectrum obtained for the $\:\text{P}\text{m}\text{A}\text{P}/\text{A}\text{P}\text{T}\text{E}\text{S}{/\text{G}\text{O}}_{\left(6.6\right)}$. At 127 °C, a weight loss of 9.78% was produced by the release of gases and water molecules adsorbed to the composite’s surface. About 33.2% of the mass was lost between 127 and 800 °C owing to the degradation of the organic component of the composite. The result shows that the produced composite is thermally stable up to the ashing temperature. On the DTA spectrum, three large derivative peaks were identified at 127, 261, and 380 °C. The data obtained show that an exothermic reaction occurred during the content phase^76^.

#### SEM, TEM, and EDX studies

**Fig. 4:**
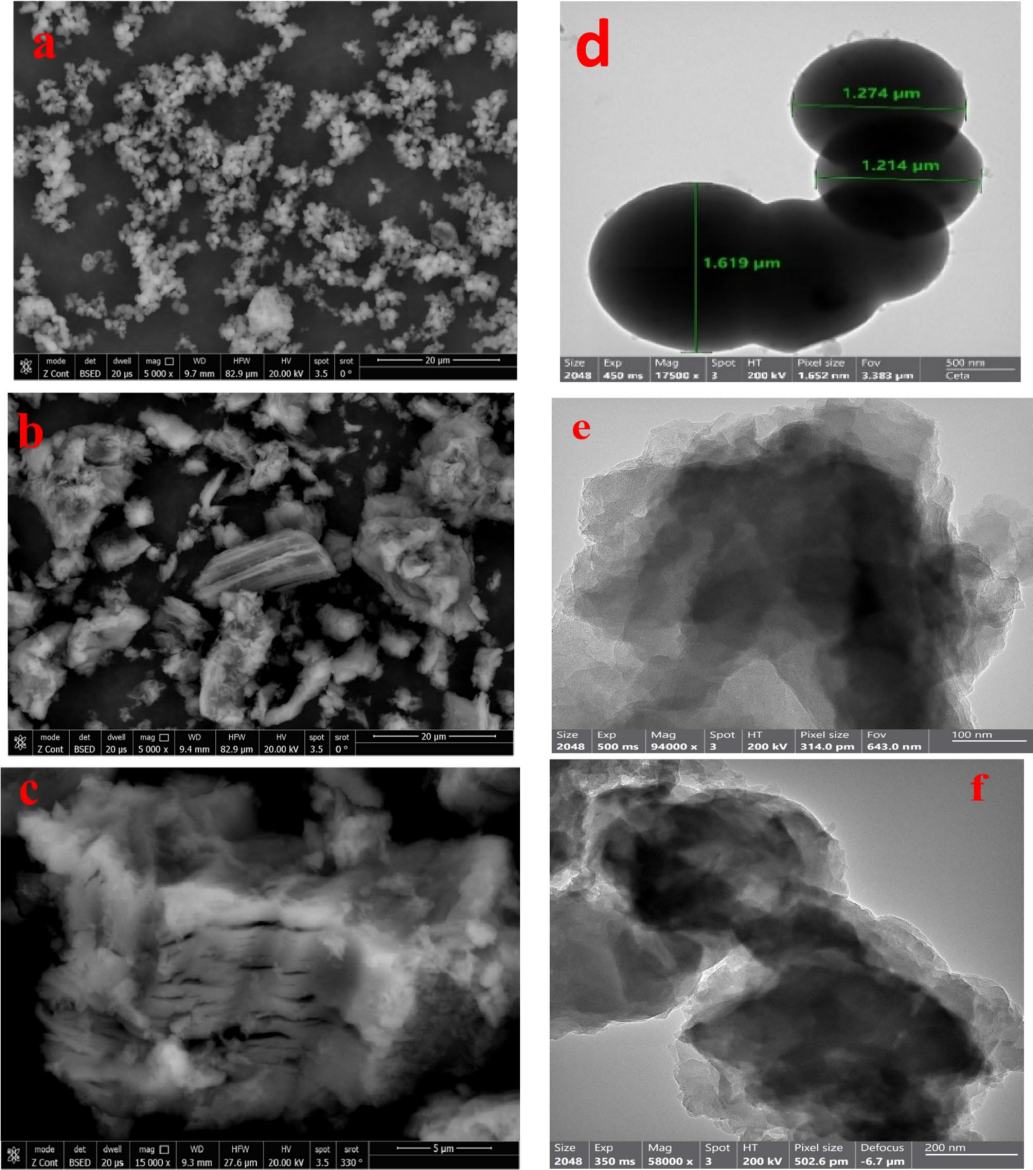
(**a**) SEM micrographs of PmAP, (**b**) SEM of $\:\text{P}\text{m}\text{A}\text{P}{/\text{G}\text{O}}_{\left(6.6\right)}$, and (**c**) SEM of $\:\text{P}\text{m}\text{A}\text{P}/\text{A}\text{P}\text{T}\text{E}\text{S}{/\text{G}\text{O}}_{\left(6.6\right)}$; (**d**) TEM micrographs of PmAP, (**e**) TEM of $\:\text{P}\text{m}\text{A}\text{P}{/\text{G}\text{O}}_{\left(6.6\right)}\:$, and (**f**) TEM of $\:\text{P}\text{m}\text{A}\text{P}/\text{A}\text{P}\text{T}\text{E}\text{S}{/\text{G}\text{O}}_{\left(6.6\right)}\:$

The morphology and the particle size of the synthesized materials were determined using SEM and TEM, respectively. Additionally, the elements of the materials and their ratio were detected from EDX charts. Figure 4a shows the SEM micrograph of PmAP, it is believed that its surface has a spherical structure overlapped with a cluster of grapes^61^. The SEM image of $\:\text{P}\text{m}\text{A}\text{P}/{\text{G}\text{O}}_{\left(6.6\right)}$ exhibits the wrapping of GO sheets around the polymer and unshaped cavities were created, Fig. 4b. The APTES treatment firmly wrapped the GO sheets around the polymer, creating slit cavities, Fig. 4c. Examining the TEM micrograms of the PmAP reveals that it has a distinct spherical plate shape with significant aggregation. The particle size has an average value of 1.28 μm^77,78^, Fig. 4d. The monomer with a hydrophilic hydroxy group and a hydrophobic aromatic ring forms micelles in water. The aggregation of such tiny monomeric micelles acts as a template during subsequent oxidation^79^. In contrast to previous reports^78^, the presence of GO in the polymerization medium eliminated these spherical plates, Fig. 4e. The dark area is due to PmAP and the transparency area indicates the formation of rGO sheets with few layers stacked with each other with fewer wrinkles as confirmed by XRD in Fig. 2^80^. The average space between the layers of rGO layers is 8–16 nm. Even after APTES treatment, the TEM micrographs of polymer-loaded GO appear as wrinkled paper with some dark areas of indeterminate shape due to PmAP, Fig. 4f. The average space between the rGO’s layers increased to 26.7–32 nm indicating the intercalation of $\:\text{P}\text{m}\text{A}\text{P}/{\text{G}\text{O}}_{\left(6.6\right)}\:$between the layers of rGO as confirmed by XRD. An EDX supplement attached to the SEM provided an indicative analysis of the in-sequence samples, Table 1. Tracing the ratios of the elements provides a preliminary assessment of the success of the GO and PmAP with APTES functionalization. Similar to its ability to form bonds with GO^69^, APTES can also form bonds with PmAP. Therefore, selecting APTES not only eliminates the polymer’s solubility but also introduces a form of incorporation between the two surfaces.

**Table 1:** EDX composition analysis of PmAP, $\:\text{P}\text{m}\text{A}\text{P}/{\text{G}\text{O}}_{\left(6.6\right)}$, and $\:\text{P}\text{m}\text{A}\text{P}/\text{A}\text{P}\text{T}\text{E}\text{S}{/\text{G}\text{O}}_{\left(6.6\right)}\:$

Sample	C wt %	*N* wt %	O wt %	Si wt %	Cl wt %	S wt %
$\:\text{P}\text{m}\text{A}\text{P}$	60.61	13.86	15.69	--	4.39	6.45
$\:\text{P}\text{m}\text{A}\text{P}{/\text{G}\text{O}}_{\left(6.6\right)}$	41.46	9.81	46.69	0.40	1.64	--
$\:\text{P}\text{m}\text{A}\text{P}/\text{A}\text{P}\text{T}\text{E}\text{S}{/\text{G}\text{O}}_{\left(6.6\right)}$	42.14	8.36	30.98	18.39	0.13	--

#### XPS

**Fig. 5:**
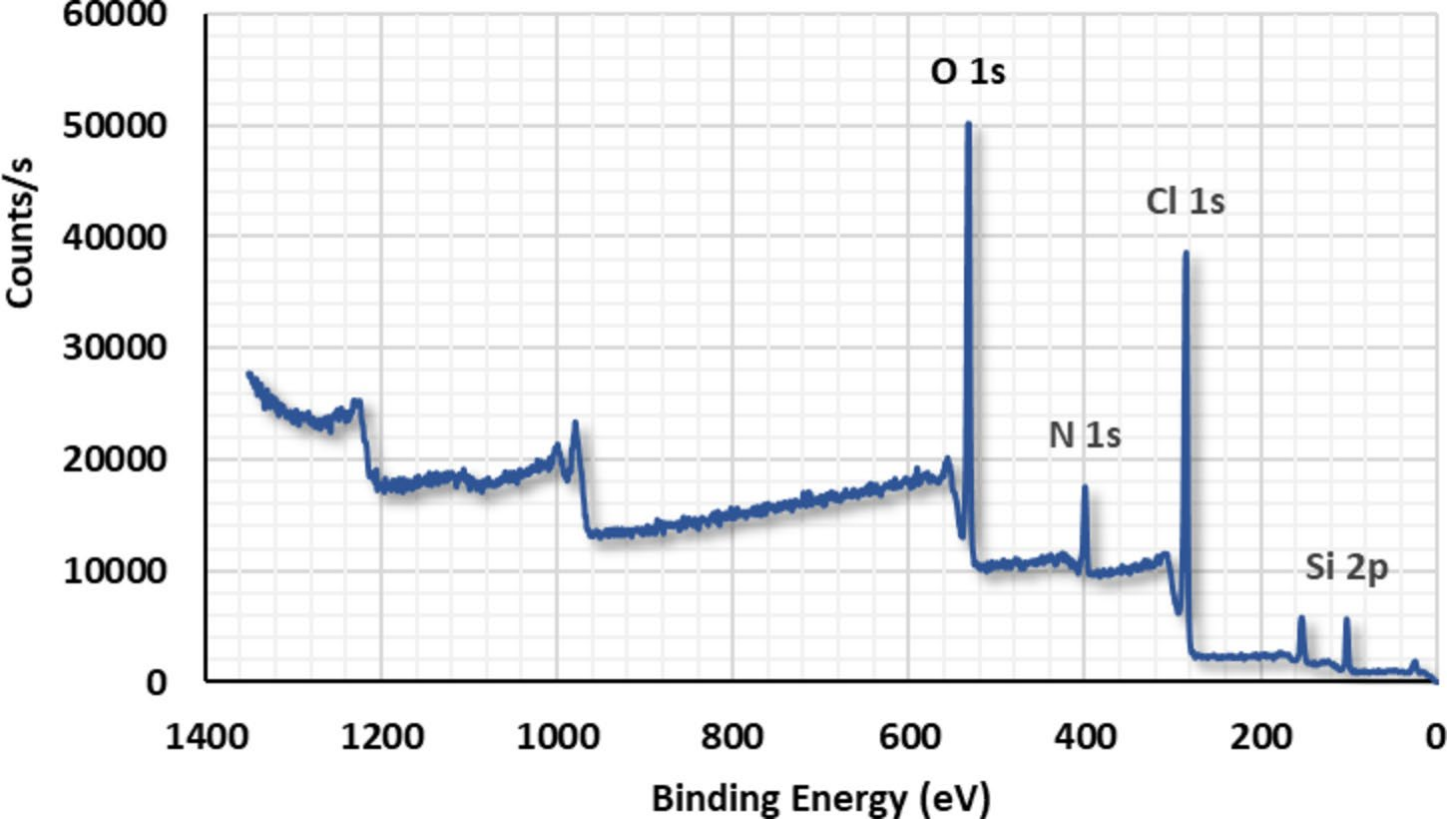
XPS spectra of $\:\text{P}\text{m}\text{A}\text{P}/\text{A}\text{P}\text{T}\text{E}\text{S}{/\text{G}\text{O}}_{\left(6.6\right)}\:$.

XPS is a method for studying the surface chemistry of a material that can detect the elemental composition, chemical, and electronic state of atoms within the material^67^. Figure 5 shows full XPS spectra and a complete composition analysis of $\:{\text{G}\text{O}}_{\left(6.6\right)}/\text{P}\text{m}\text{A}\text{P}/\text{A}\text{P}\text{T}\text{E}\text{S}$. An element analysis comparable to EDX was obtained, although EDX is not quantitative but rather indicative. XPS elemental analysis confirms the presence of Si2p that belongs to APTES, Table 2.

**Table 2:** Complete composition analysis of $\:\text{P}\text{m}\text{A}\text{P}/\text{A}\text{P}\text{T}\text{E}\text{S}{/\text{G}\text{O}}_{\left(6.6\right)}$

	Peak BE	FWHM (eV)	Area (*P*)	Atomic %
O1s	532.5	3.740	158,983	24.53
C1s	285.5	4.010	151,887	59.80
N1s	400.2	4.000	35,715	8.300
Si2p	102.9	3.270	16,643	7.370

### Adsorption studies

**Fig. 6:**
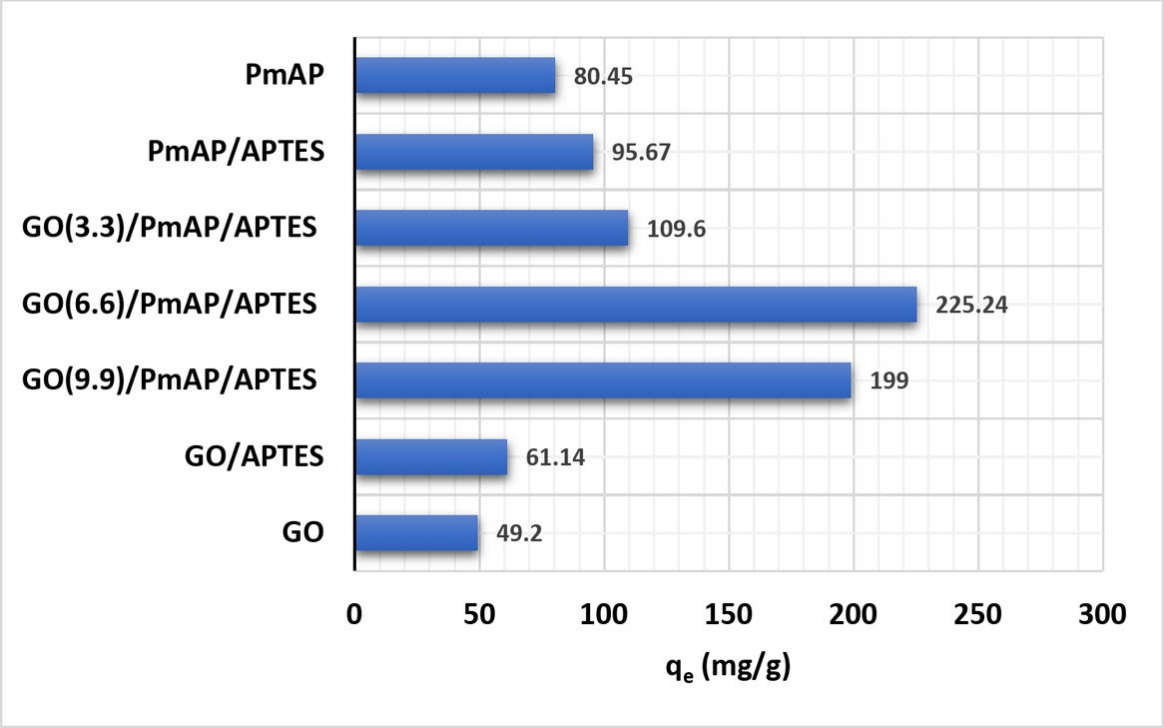
The adsorption of Cu(II) ions by GO, GO/APTES, PmAP, $\:\text{P}\text{m}\text{A}\text{P}/\text{A}\text{P}\text{T}\text{E}\text{S}{/\text{G}\text{O}}_{\left(6.6\right)}:\:\:$Cu(II) ions initial concentration 300 mg/L, adsorbent dosage 0.01 g, pH = 7, temperature 25 °C, stirring speed 120 rpm, contact time 3 h.

The concentration of GO in composite $\:\text{P}\text{m}\text{A}\text{P}/\text{A}\text{P}\text{T}\text{E}\text{S}{/\text{G}\text{O}}_{\left(\text{x}\right)}$ had a strong effect on the adsorption capacity of the composite towards Cu(II). The adsorption of Cu(II) on three composites, namely the $\:\text{P}\text{m}\text{A}\text{P}/\text{A}\text{P}\text{T}\text{E}\text{S}{/\text{G}\text{O}}_{\left(3.3\right)}$, $\:\text{P}\text{m}\text{A}\text{P}/\text{A}\text{P}\text{T}\text{E}\text{S}{/\text{G}\text{O}}_{\left(6.6\right)}$ and the $\:\text{P}\text{m}\text{A}\text{P}/\text{A}\text{P}\text{T}\text{E}\text{S}{/\text{G}\text{O}}_{\left(9.9\right)}$ were studied under the same experimental conditions. Additionally, the adsorption behavior of unmodified GO, GO/APTES, PmAP and PmAP/APTES was investigated as a means of comparison, Fig. 6. The findings indicated that treating GO with PmAP greatly boosted its Cu(II) adsorption capability. The role of APTES is to prevent the dissociation of PmAP and its fixation on the surface of GO. Moreover, it provides the surface with free amine group sites. The enhanced Cu(II) adsorption was attributed to the increase in the composite’s chelation sites due to the rising GO concentration and increasing surface area. However, the adsorption capacity of $\:\text{P}\text{m}\text{A}\text{P}/\text{A}\text{P}\text{T}\text{E}\text{S}{/\text{G}\text{O}}_{\left(6.6\right)}\:$was less than that of $\:\text{P}\text{m}\text{A}\text{P}/\text{A}\text{P}\text{T}\text{E}\text{S}{/\text{G}\text{O}}_{\left(6.6\right)}$. This drop might result from PmAP aggregation providing less surface area and nonporosity as confirmed by N_2_ adsorption-desorption measurements. As a result, the $\:\text{P}\text{m}\text{A}\text{P}/\text{A}\text{P}\text{T}\text{E}\text{S}{/\text{G}\text{O}}_{\left(6.6\right)}$ composite was chosen as a model for further exploration.

#### Optimizing solution pH

**Fig. 7:**
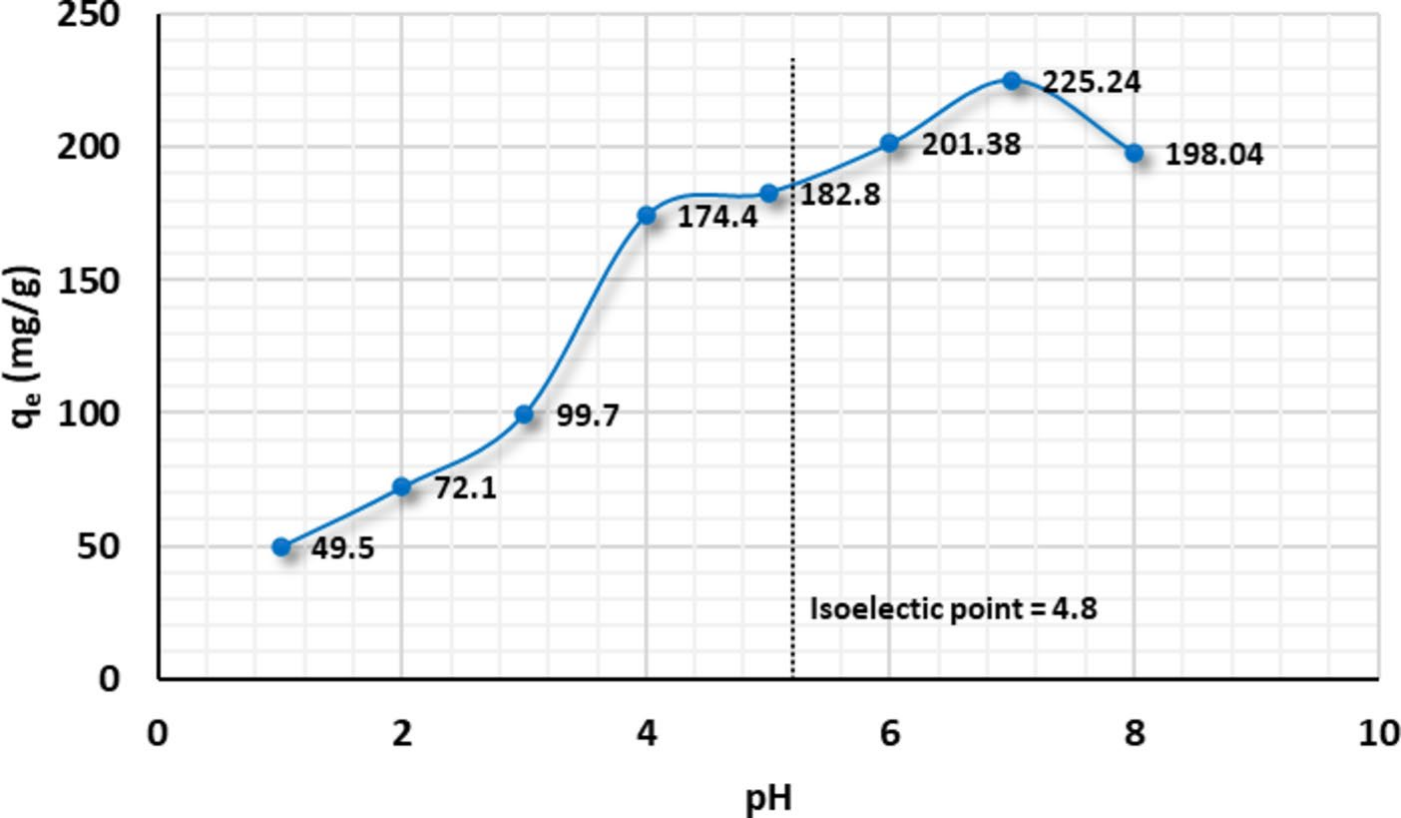
Effect of solution pH on adsorption capacity of Cu(II) ions onto $\:\text{P}\text{m}\text{A}\text{P}/\text{A}\text{P}\text{T}\text{E}\text{S}{/\text{G}\text{O}}_{\left(6.6\right)}$: Cu(II) ions initial concentration 300 mg/L, adsorbent dosage 0.01 g, temperature 25 °C, stirring speed 120 rpm, contact time 3 h.

Solution pH is critical in adsorption; it affects the adsorbent’s surface charge, contaminants ionization, and species type. Herein, the relation between the initial pH and the adsorption capacity was investigated, Fig. 7. The pH was adjusted for the desired value from 1 to 8, using the universal buffer. Cu(II) adsorption capacity rose as the pH of the solution increased from 1 to 7. This behavior might be explained by the adsorbent’s surface charge versus the adsorbate’s predominant species. The $\:\text{P}\text{m}\text{A}\text{P}/\text{A}\text{P}\text{T}\text{E}\text{S}{/\text{G}\text{O}}_{\left(6.6\right)}\:$composite surface is coated with hydroxyl, carboxyl, and amino groups that take diverse forms at different pH levels. The isoelectric point of the composite surface was recorded at 4.8 (dotted line). When the pH of the $\:\text{P}\text{m}\text{A}\text{P}/\text{A}\text{P}\text{T}\text{E}\text{S}{/\text{G}\text{O}}_{\left(6.6\right)}$medium was lower than the isoelectric point, the amine groups on the surface were protonated. The acquired positive charge resulted in an electrostatic repulsion with the positively charged Cu(II) species which is the dominant species at pH < 6.0^81,82^. As pH increases, the proton competition deteriorates as the concentration of $\:{\text{H}}^{+}$ decreases. As a result, the equilibrium adsorbed amount of Cu(II) ion increased. Noting that the increase in adsorption rate was attenuated in the range of 4 to 6 as a result of the hybrid’s surface charge transformation. At a pH of 7, the composite showed the maximum adsorption behavior. As the pH increased, the adsorption deteriorated as the Cu(II) ion became hydrolysis and produced a secondary reaction product such as Cu(OH)_2_, this can decrease the removal efficiency directly^83^.

#### Optimizing contact time

**Fig. 8:**
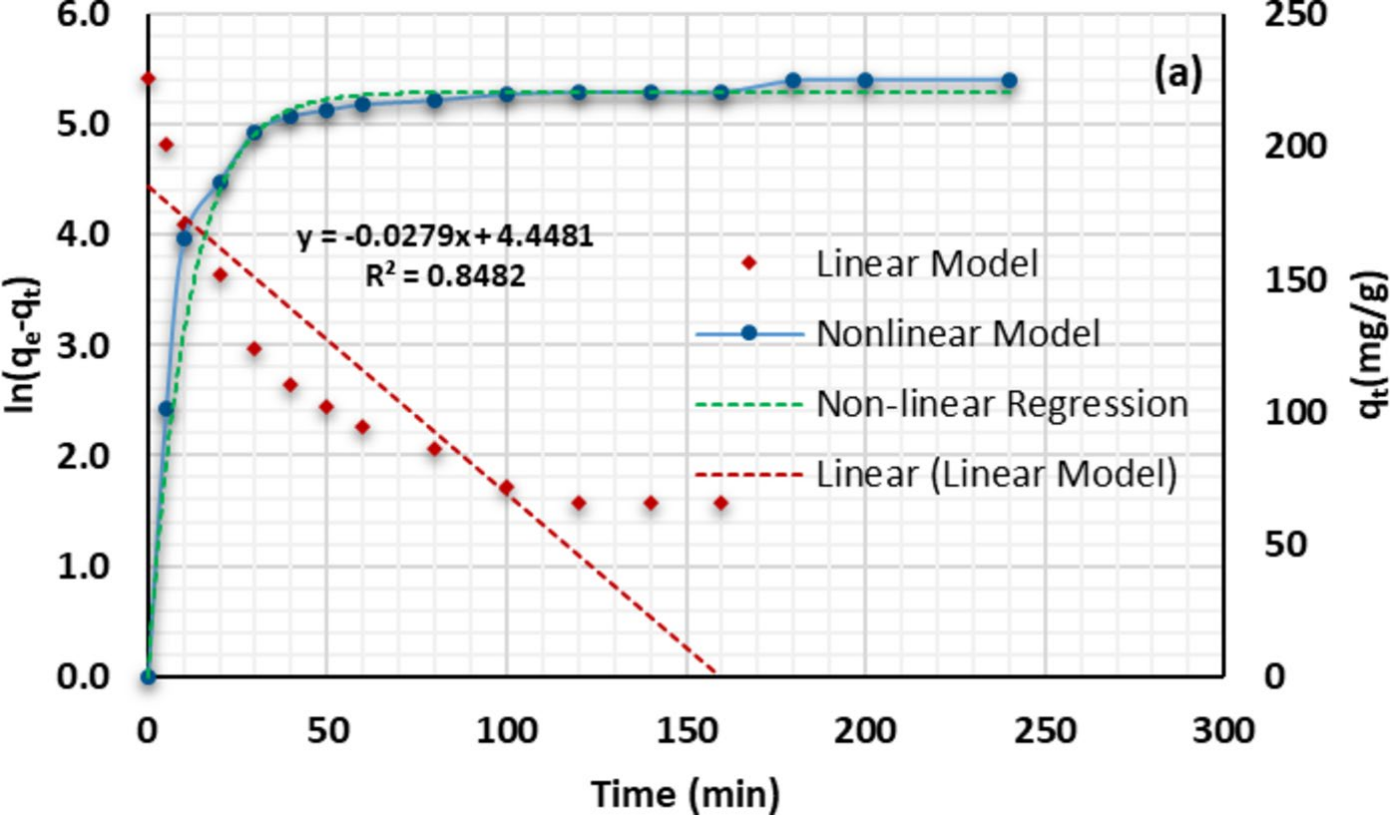
Effect of contact time on the adsorption data of Cu(II) ions onto $\:\text{P}\text{m}\text{A}\text{P}/\text{A}\text{P}\text{T}\text{E}\text{S}{/\text{G}\text{O}}_{\left(6.6\right)}$. [Cu(II)]_o_=150 mg/L, adsorbent dosage 0.01 g, temperature 25 °C, stirring speed 120 rpm, contact time 4 h.

To optimize contact time, Cu(II) uptake was investigated at time intervals ranging from 1 to 240 min using an adsorbent dose of 0.01 g, solution pH of 7, and initial Cu(II) ion concentration of 150 mg/L. The obtained results indicated that the adsorption of Cu(II) ions onto the composite is multiphase. In the first phase, the adsorption increased rapidly during the initial 50 min of the experiment and then gradually increased from 50 to 240 min., Fig. 8. After 240 min., the adsorption capacity has almost nonchanged. The adsorption rate was initially high due to the accessible free sites on the composite. However, the gradual increase in the second phase was caused by Cu(II) diffusion from the outer layer to the inner pores^84^. At equilibrium time, adsorption capacity shows a plateau owing to the saturation of adsorption sites on the adsorbent surface^85,86^.

#### Adsorption kinetics

Pseudo-first-order (PFO) and pseudo-second-order (PSO) kinetic models were used to calculate the Cu(II) ions removal rate by the $\:\text{P}\text{m}\text{A}\text{P}/\text{A}\text{P}\text{T}\text{E}\text{S}{/\text{G}\text{O}}_{\left(6.6\right)}$ composite. Figure S4 a and b depict the linear and nonlinear fitting of PFO and PSO, respectively, of the obtained data. Table 3 contains a list of the two kinetic models’ adsorption parameters and their correlation coefficients. The Cu(II) scavenging onto $\:\text{P}\text{m}\text{A}\text{P}/\text{A}\text{P}\text{T}\text{E}\text{S}{/\text{G}\text{O}}_{\left(6.6\right)}$is much closer to the PSO kinetic model where $\:{\text{R}}^{2}=0.999$ in both linear and nonlinear orthogonal distance regression. However, in the case of PFO, there is a significant difference between the outcomes of linear and nonlinear regression. Also, in the perplexing case of PFO non-linear regression with R^2^ = 0.999, the reduced chi-square value was 10.31, compared to 2.74 for PSO. Furthermore, the estimated adsorption quantities (q_e, est_ )for the PSO equation were almost identical to the experimental adsorption capacity (q_e, exp_). These findings suggest that Cu(II) scavenging onto $\:\text{P}\text{m}\text{A}\text{P}/\text{A}\text{P}\text{T}\text{E}\text{S}{/\text{G}\text{O}}_{\left(6.6\right)}$is much closer to the PSO kinetic model^84,86^. This data implies that the adsorption process was chemical in nature. This is anticipated due to the proliferation of functional groups on the adsorbent surface capable of coordinating/exchanging electrons with the Cu(II) ions^81^.

**Table 3:** Kinetic parameters for the adsorption of Cu(II) ions onto $\:\text{P}\text{m}\text{A}\text{P}/\text{A}\text{P}\text{T}\text{E}\text{S}{/\text{G}\text{O}}_{\left(6.6\right)}$composite

Model		q_e,exp_ (mg/g)	Parameters			
			q_e,est_ (mg/g)	k_1_ (1/min)	R^2^	χ^2^
PFO	Linear	224.6	85.46	$\:0.028$	0.848	
	Nonlinear		$\:220.7\pm\:1.06$	$\:0.090\pm\:0.01$	0.999	10.31

		q_e,exp_ (mg/g)	q_e,est_ (mg/g)	k_2_ (g/mg min)	R^2^	χ^2^
PSO	Linear	224.6	227.3	$\:1.21\text{*}{10}^{-3}$	0.999	
	Nonlinear		$\:228.0\pm\:0.85$	$\:1.21\times\:{10}^{-3}\pm\:8.95\times\:{10}^{-5}$	0.999	2.740

#### Optimizing Cu(II) initial concentration

Figure S5 depicts the effect of Cu(II) initial concentration on the removal efficiency and adsorption capacity of the synthesized $\:\text{P}\text{m}\text{A}\text{P}/\text{A}\text{P}\text{T}\text{E}\text{S}{/\text{G}\text{O}}_{\left(6.6\right)}$. As the initial Cu(II) concentration increased from 75 to 300 mg/L, the removal efficiency decreased from 99.23 to 36.99%. Decreasing efficiency was attributed to the saturation of the accessible active sites for adsorption. Whereas the rise in initial Cu(II) concentration was followed by an increase in the number of ions loaded on the adsorbent’s surface. At an initial Cu(II) concentration of 300 mg/L, the adsorption capacity reached 221.96 mg/g as the Cu(II) ions became available in abundance to the binding sites of the adsorbent. The initial concentration of Cu(II) ions was determined to be 150 mg/L, which also allows the study of the equilibrium state.

#### Adsorption isotherms models

Adsorption isotherms are mathematical equations expressing the connection between adsorbate concentrations and the amount of adsorption at any temperature. Langmuir, Freundlich, and Dubinin-Radushkevich (D–R) isotherm models were used to analyze the adsorption behavior of the synthesized $\:\text{P}\text{m}\text{A}\text{P}/\text{A}\text{P}\text{T}\text{E}\text{S}{/\text{G}\text{O}}_{\left(6.6\right)}$for Cu(II) at three different temperatures. Errors generated during linearization result in inaccurate parameter estimations. Therefore, linear and nonlinear regressions were used instead to calculate the parameters^87^ and tabulated in Table 4. By comparing the correlation coefficient, R^2^, it was found that the compatibility of Freundlich and Langmuir isotherms with experimental data. Freundlich intensity factor (1/n) was lower than unity and increased with temperature but still lower than unity, suggesting that the uptake of Cu(II) on $\:\text{P}\text{m}\text{A}\text{P}/\text{A}\text{P}\text{T}\text{E}\text{S}{/\text{G}\text{O}}_{\left(6.6\right)}$is favorable^88^. However, the K_F_ values increase with increasing temperature. This suggests that the adsorption process is endothermic and favorable at higher temperatures^89^. The Langmuir maximum adsorption capacity, q_max_ increased with increasing temperature, implying this adsorption may be an endothermic process. Furthermore, the separation factor (R_L_), characteristics of the Langmuir isotherm, has values in the range of 0.008–0.044, implying that the adsorption is a favorable process. The D-R model provides information about the mean sorption energy, $\:{\text{E}}_{D},$ which predicts the type of adsorption on the surface. The values of $\:{\text{E}}_{D}$ were more than 16 kJ/mol at all experimental temperatures indicating that the adsorption was chemisorption^90^. Additionally, its value declines as the temperature rises, but is still higher than 16 kJ/mol. Langmuir’s maximum adsorption capacity (q_max_) was compared with that of different adsorbents reported in the literature and listed in Table 5. $\:\text{P}\text{m}\text{A}\text{P}/\text{A}\text{P}\text{T}\text{E}\text{S}{/\text{G}\text{O}}_{\left(6.6\right)}$ has high uptake capacity for Cu(II) ions.

**Table 4:** Isotherm parameters and their correlation coefficient (R^2^) for the adsorption of Cu(II) onto $\:\text{P}\text{m}\text{A}\text{P}/\text{A}\text{P}\text{T}\text{E}\text{S}{/\text{G}\text{O}}_{\left(6.6\right)}$

T (K)	Langmuir isotherm			Freundlich isotherm			D-R isotherm		
	q_max_ (mg/g)	K_L_ (L/mg)	R^2^	K_F_ (mg/g)	1/n	R^2^	q_m_ (mg/g)	*E* (kJ/mol)	R^2^
Linear form									
298	227.3	0.288	0.998	167.0	0.054	0.983	214.0	26.73	0.924
303	256.4	0.394	0.999	177.7	0.072	0.991	243.6	31.62	0.924
308	294.1	0.420	0.998	185.2	0.096	0.994	276.7	22.36	0.969
313	322.6	0.373	0.998	191.6	0.106	0.993	293.5	22.36	0.957
Non-linear form									
293	227.3	0.195	0.995	143.3	0.087	0.999			
303	264.7	0.175	0.998	177.5	0.072	0.980			
308	296.8	0.267	0.999	207.5	0.069	0.999			
313	324.5	0.209	0.998	193.8	0.103	0.986			

**Table 5:** Adsorption comparison between recent adsorbents for Cu(II) ions in the literature.

Adsorbent	$\:{{q}_{\:max}}_{\:}\:$(mg/g)	Ref.
GO/PEI	150.9	^82^
GO/PVA	247.16	^85^
Sr-GO/montmorillonite	101.83	^110^
GO/silica@PANI (30–50 °C)	258.3–314.1	^84^
EDTA functionalized magnetic GO	301.2	^111^
A mesoporous guanidine functionalized Santa Barbara Amorphous-15 (SBA-15)/Fe_3_O_4_	344.82	^112^
PEI-modified carboxymethyl chitosan	175.56	^98^
MgAl-layered double hydroxide nanosheets assembled on GO	80.72	^97^
poly(allylamine hydrochloride) (PAH)/GO	349.03	^11^
pyrazole based chitosan Schiff base material/Fe_3_O_4_/MoS_2_	125	^113^
MnO2/polyethyleneimine/tannic acid,	124.5	^114^
GO_(6.6)_/PmAP/APTES.	324.54	This work

#### Adsorption thermodynamics

The thermodynamic parameters are evaluated using the Van’t Hoff equation (Eq. 1) and Eq. (2) by introducing the experimental data at four different temperatures into these equations. The calculated thermodynamic parameters are listed in Table 6. The positive values of the enthalpy ($\:{\Delta\:}\text{H}=78.39\:\text{k}\text{J}/\text{m}\text{o}\text{l}$) indicate that the removal process of Cu(II) was endothermic^91^. Moreover, the magnitude of the $\:{\Delta\:}\text{H}^\circ\:$ was large enough to consider the adsorption process is chemosorption in nature. Positive entropy values $\:{\Delta\:}\text{S}^\circ\:$ indicate accumulating randomness at the solid-liquid interface during adsorption. However, its small value limits the system disorder that arises from the adsorption of Cu(II) ions^92^. The negative sign of $\:{\Delta\:}\text{G}^\circ\:$ indicates a spontaneous adsorption process. The gradual decrease in $\:{\Delta\:}\text{G}^\circ\:$ from − 1.694 to -5.552 kJ/mol as temperature increases from 298.15 to 313.15 °K is a plausible explanation for the increase in sorption capacity with temperature^93,94^.

**Table 6:** Thermodynamic parameters for Cu(II) sorption on $\:\text{P}\text{m}\text{A}\text{P}/\text{A}\text{P}\text{T}\text{E}\text{S}{/\text{G}\text{O}}_{\left(6.6\right)}$

$\:{\Delta\:}\text{H}^\circ\:\:\text{k}\text{J}/\text{m}\text{o}\text{l}$	$\:{\Delta\:}\text{S}^\circ\:\:\:\text{k}\text{J}/\text{K}.\text{m}\text{o}\text{l}$	$\:{\Delta\:}\text{G}^\circ\:,\:\text{k}\text{J}/\text{m}\text{o}\text{l}$			
		298.15 °K	303.15 °K	308.15 °K	313.15 °K
78.39	0.268	− 1.694	− 2.745	− 4.586	− 5.552

1 $$\:\text{ln}{\text{K}}_{\text{d}}=\:-\frac{\varDelta\:{\text{H}}_{\text{a}\text{d}\text{s}}}{\text{R}\text{T}}+\frac{{\varDelta\:\text{S}}_{\text{a}\text{d}\text{s}}}{\text{R}}$$

2 $$\:\varDelta\:{\text{G}}_{\text{a}\text{d}\text{s}}=\varDelta\:{\text{H}}_{\text{a}\text{d}\text{s}}-\text{T}\varDelta\:{\text{S}}_{\text{a}\text{d}\text{s}}\:$$

Where; K_d_ is the distribution coefficient (K_d_ = q_e_/C_e_).

#### Adsorption mechanism

**Fig. 9:**
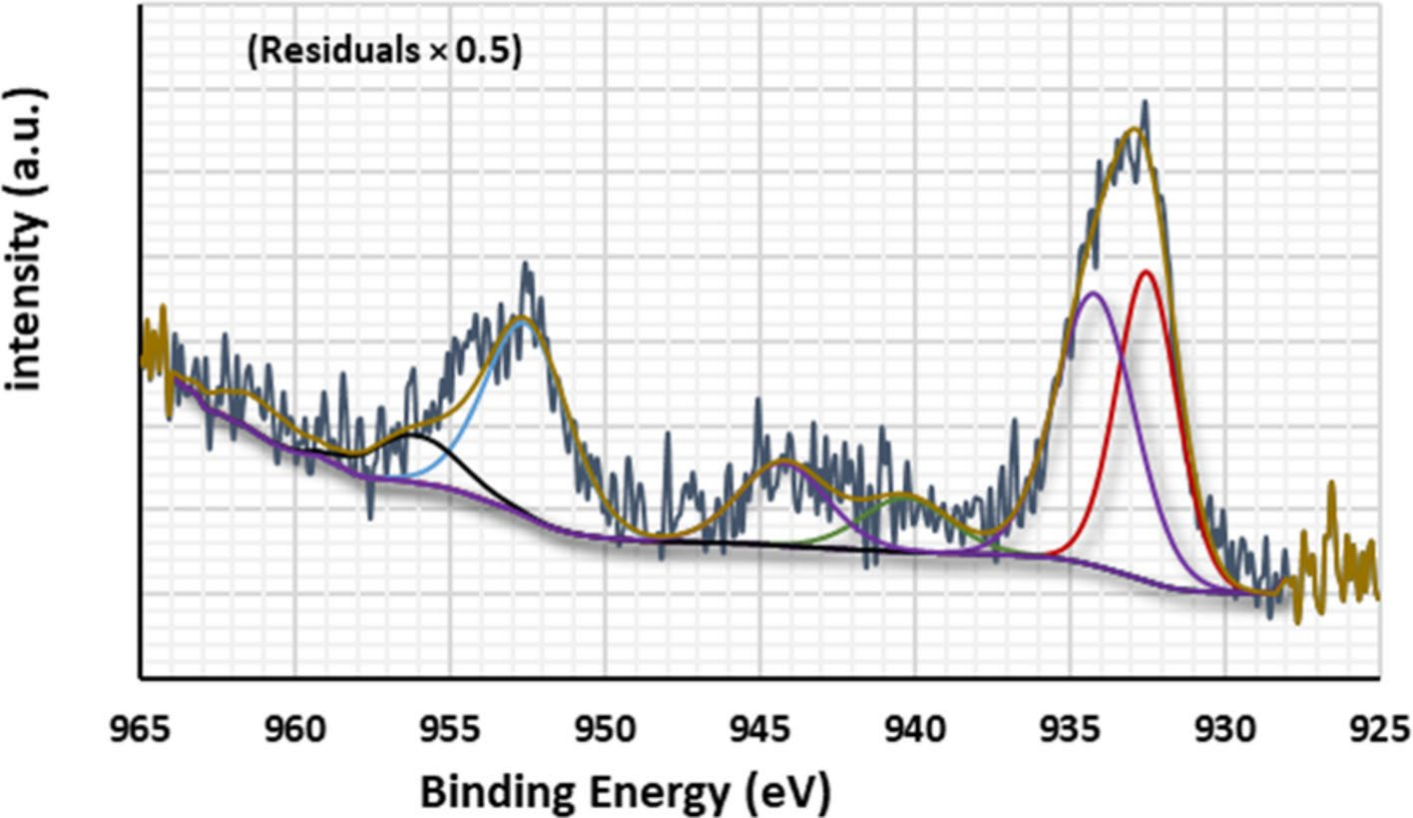
The XPS survey spectrum of the Cu signal peaks appeared after adsorption onto $\:\text{P}\text{m}\text{A}\text{P}/\text{A}\text{P}\text{T}\text{E}\text{S}{/\text{G}\text{O}}_{\left(6.6\right)}$.

**Fig. 10:**
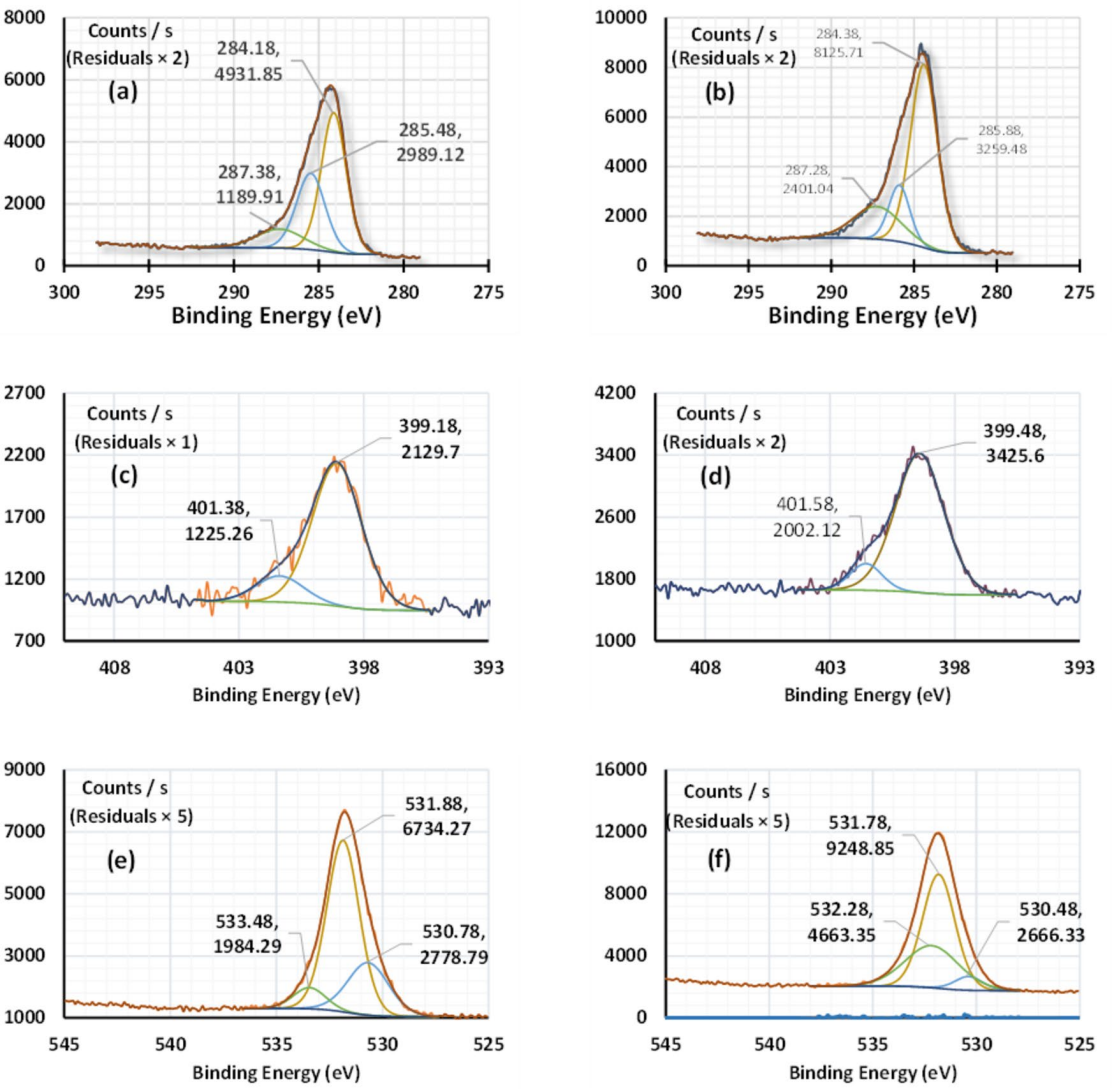
The high-resolution XPS spectra of C1s, N1s, and O1s obtained before (**a**, **c**, and **e**) and after (**b**, **d**, and **f**) Cu(II) ions sorption on $\:\text{P}\text{m}\text{A}\text{P}/\text{A}\text{P}\text{T}\text{E}\text{S}{/\text{G}\text{O}}_{\left(6.6\right)}$.

The XPS survey spectrum of the Cu appeared after adsorption onto $\:\text{P}\text{m}\text{A}\text{P}/\text{A}\text{P}\text{T}\text{E}\text{S}{/\text{G}\text{O}}_{\left(6.6\right)}$ with a list of the signal peaks shown in Fig. 9. The characteristic peak at 932.49 eV with an atomic ratio of 24.80% was assigned for Cu(I) chloride. Moreover, the main XPS peak at 956.08 eV with an atomic ratio of 5.59% gave evidence for the existence of Cu NPs ($\:{\text{C}\text{u}}^{0}$). This might suggest that the Cu(II) ions undergo reduction within adsorption. Cu NPs stabilization arose mainly through the interaction between hydroxyl groups on the surface of GO and Cu^0^ ($\:\text{O}-{\text{C}\text{u}}^{0}$ interactions)^95^. The presence of two strong shake-up satellite peaks at 940.27- 944.26 eV, and 960.18-962.58 eV is also evidence of the presence of Cu(II) as well^96^. The signal peak at 934.18 with an atomic ratio of 28.77% suggests that Cu(II) ions were linked through oxygen during adsorption e.g. surface–OH–Cu(II) and/or surface–O–Cu(II)^97^. The high-resolution spectra of C1s, N1s, and O1s before and after Cu(II) uptake were shown in Fig. 10. The C1s spectrum of $\:\text{P}\text{m}\text{A}\text{P}/\text{A}\text{P}\text{T}\text{E}\text{S}{/\text{G}\text{O}}_{\left(6.6\right)}$before sorption, Fig. 10a was fitted to three peaks at 284.12, 285.47, and 287.31 eV, which were assigned to C = C, C–C/H, and C = O/N, respectively^98^. After Cu(II) adsorption, Fig. 10b, the first two peaks shifted to the higher binding energies of 284.41 and 285.92 eV, while the last peak shifted to the lower binding energy of 286.59 eV. Notably, there is an increase in the atomic ratio of C = C and C = O versus a decrease in C-C/C-H. This means that the adsorption surface changed into an oxidized state^99^.

The N1s spectrum of $\:\text{P}\text{m}\text{A}\text{P}/\text{A}\text{P}\text{T}\text{E}\text{S}{/\text{G}\text{O}}_{\left(6.6\right)}$before sorption, Fig. 10c was fitted to two peaks at 399.08 and 401.38 eV, which were assigned to (-N-) and (= N-), respectively^100–102^. After Cu(II) adsorption, Fig. 10d, these peaks shifted to the higher binding energy of 399.42 and 401.57 eV verifying the reduction of the nitrogen charge density^98^. The atomic percentage of (-N-) increased from 84.88 to 88.87%, while that of (= N-) decreased from 15.12 to 11.13%. Moreover, a peak-to-peak height changed from 1158.94 to 417.16 to 1785.22 and 599.97 CPS, respectively. The significant decrease in the quinoid imine ratio (= N-), in contrast to the benzenoid amine ratio (-N-) might be responsible for the redox reaction involving the reduction Cu(II) to Cu^0^ upon adsorption^103–106^.

The O1s spectrum of $\:\text{P}\text{m}\text{A}\text{P}/\text{A}\text{P}\text{T}\text{E}\text{S}{/\text{G}\text{O}}_{\left(6.6\right)}$before sorption, Fig. 10e was fitted to three peaks at 530.68, 531.86, and 533.44 eV, which were assigned to O-H, C = O, and C-O, respectively. After Cu(II) adsorption, Fig. 10f, the first two peaks shifted to the lower binding energies of 530.36, and 531.81 eV as the adsorbent transformed to the oxidized state. In addition, the atomic percentage of C = O and –OH were dramatically decreased indicating that the abundant –OH and –COOH on $\:\text{P}\text{m}\text{A}\text{P}/\text{A}\text{P}\text{T}\text{E}\text{S}{/\text{G}\text{O}}_{\left(6.6\right)}$might contribute 2 or 3 neighboring oxygen atoms to create COO–Cu and –O–Cu^98,107^.

#### Reusability and recycling

**Fig. 11:**
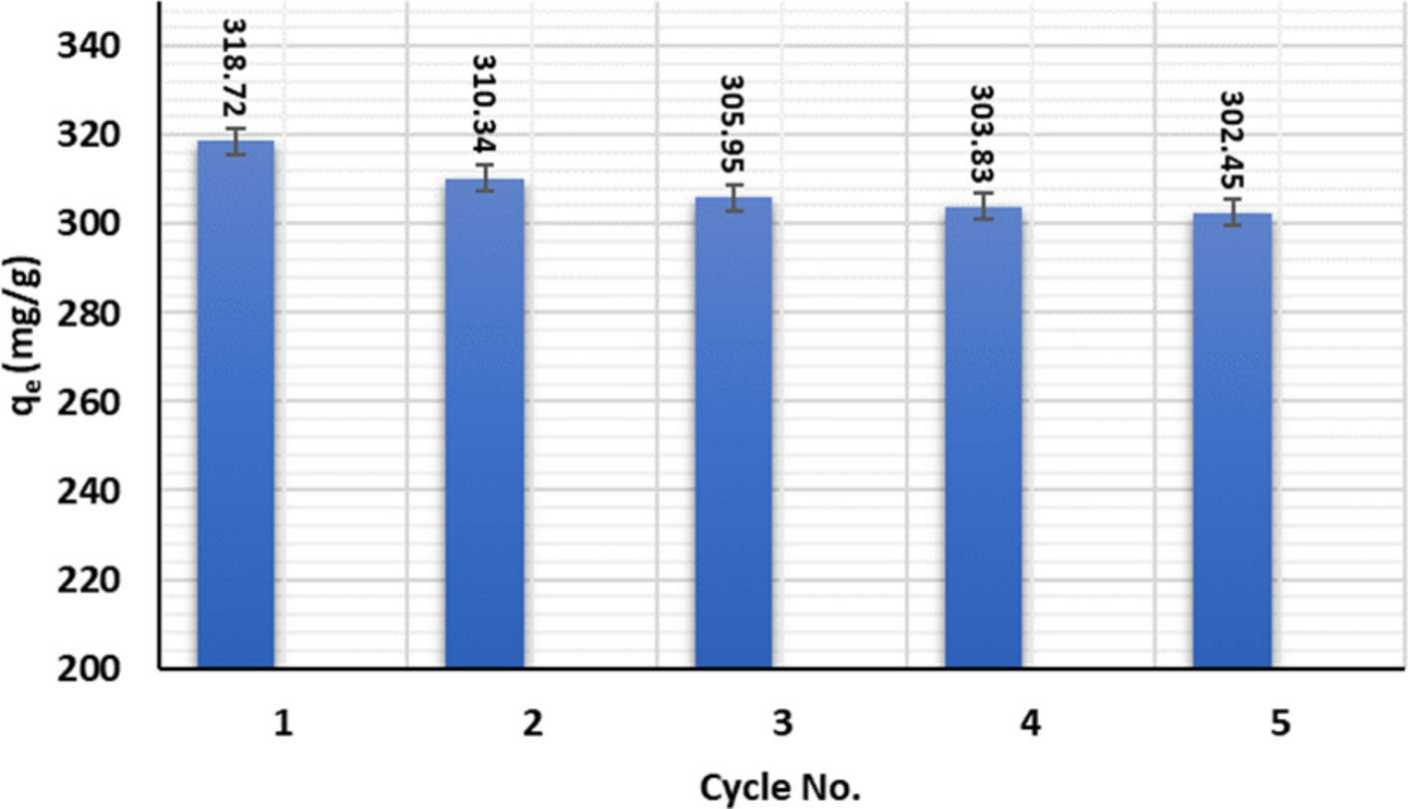
A bar chart with the standard error represents sorption-desorption cycles of the adsorbed Cu(II) onto $\:\text{P}\text{m}\text{A}\text{P}/\text{A}\text{P}\text{T}\text{E}\text{S}{/\text{G}\text{O}}_{\left(6.6\right)}$. [Cu(II)]_o_ =150 mg/L, adsorbent dosage 0.01 g, pH = 7, and stirring speed 120 rpm.

The reusability of the adsorbent is a significant indication of the adsorbent’s cost-effectiveness in practical applications. After Cu(II) adsorption, consecutive acidic and alkaline media were used to revive the spent adsorbent. The spent adsorbent was soaked for 2 h at pH = 1 for regeneration before changing the medium to alkaline using the 0.1 NaOH solution. The regenerated adsorbent was washed frequently with demineralized water before drying. After 5 cycles, the Cu(II) adsorption capacity was maintained at about 93.7%, Fig. 11. The minor reduction in adsorption was attributable to the creation of permeant bonds with the surface, which was entirely difficult to eliminate. The use of an alkaline medium aided in the precipitation of the adsorbent’s released Cu(II). Furthermore, it eliminates the deterioration of the adsorption capacity that ceased when the activation process was completed. The results showed that $\:\text{P}\text{m}\text{A}\text{P}/\text{A}\text{P}\text{T}\text{E}\text{S}{/\text{G}\text{O}}_{\left(6.6\right)}$is a good adsorbent that could be used to get rid of heavy metals.

## Conclusion

In this study, composites $\:\text{P}\text{m}\text{A}\text{P}/\text{A}\text{P}\text{T}\text{E}\text{S}{/\text{G}\text{O}}_{\left(\text{x}\right)}$were fabricated where x is the initial GO concentration in the polymerization medium (mg/mL). The complete characterization of the prepared $\:\text{P}\text{m}\text{A}\text{P}/\text{A}\text{P}\text{T}\text{E}\text{S}{/\text{G}\text{O}}_{\left(6.6\right)}$ composite was done. $\:\text{P}\text{m}\text{A}\text{P}/\text{A}\text{P}\text{T}\text{E}\text{S}{/\text{G}\text{O}}_{\left(6.6\right)}$ showed the highest surface area and mesoporosity. Cu(II) is rapidly adsorbed on $\:\text{P}\text{m}\text{A}\text{P}/\text{A}\text{P}\text{T}\text{E}\text{S}{/\text{G}\text{O}}_{\left(6.6\right)}$ and the pseudo-second-order model well describes the sorption kinetics. Langmuir and Freundlich models accurately fit the sorption isotherms. The sorption process is endothermic and spontaneous, as shown by the thermodynamic data. $\:\text{P}\text{m}\text{A}\text{P}/\text{A}\text{P}\text{T}\text{E}\text{S}{/\text{G}\text{O}}_{\left(6.6\right)}\:$composites are efficient adsorbents for the removal of Cu(II) ions from large volumes of aqueous solutions with an adsorption capacity of 324.536 ± 12.044 mg/g at 40 °C and pH 7. The interaction of Cu(II) with the functional groups on the surface demonstrates that Cu(II) ions might be reduced upon adsorption with subsequent oxidation of the polymeric constituent. Overall, the multifunctional groups on the prepared composite represent a promising alternative, a more effective adsorbent surface for removing many pollutants from wastewater, such as heavy metals and dyes, with excellent adsorption capacity.

## Experimental

### Materials

The chemicals involved in this study were from an analytical grade received from prime suppliers. Their purity indicated on the package was sufficient and used without further purification. The chemicals used, suppliers, chemical formula, molecular weight, and assay are listed in Table 7.

**Table 7:** The chemicals used with a brief overview of chemical properties.

Chemicals	Supplier	Formula	M.wt (g/mol)	Assay (%)
Potassium dihydrogen phosphate	UNIVERSAL	$\:\text{K}{\text{H}}_{2}\text{P}{\text{O}}_{4}$	174.18	98
Dipotassium hydrogen phosphate	UNIVERSAL	$\:{\text{K}}_{2}\text{H}\text{P}{\text{O}}_{4}$	136.09	99
Sodium hydroxide	Riedel-de Haën	$\:\text{N}\text{a}\text{O}\text{H}$	40	99.8
3-Aminopropyl triethoxysilane (APTES)	Acros Organics	$\:{\text{H}}_{2}\text{N}{\left(\text{C}{\text{H}}_{2}\right)}_{3}\text{S}\text{i}{\left(\text{O}{\text{C}}_{2}{\text{H}}_{5}\right)}_{3}$	221.37	≥ 98
Graphite	LOBA Chemie	--	12.01	98%
Orthophosphoric acid	SDFCL	$\:{\text{H}}_{3}\text{P}{\text{O}}_{4}$	98	85
Sulfuric acid	ADWIC	$\:{\text{H}}_{2}\text{S}{\text{O}}_{4}$	98.08	98
Potassium permanganate	ADWIC	$\:\text{K}\text{M}\text{n}{\text{O}}_{4}$	158.034	99
Hydrochloric acid	SDFCL	$\:\text{H}\text{C}\text{l}$	36.458	30–34%
3-aminophenol	LOBA Chemie	$\:{\text{C}}_{6}{\text{H}}_{7}\text{N}\text{O}$	109.13	99
Sodium acetate	ADWIC	$\:\text{C}{\text{H}}_{3}\text{C}\text{O}\text{O}\text{N}\text{a}$	82.034	99
Ammonium persulfate (APS)	LOBA Chemie	$\:{\left(\text{N}{\text{H}}_{4}\right)}_{2}{\text{S}}_{2}{\text{O}}_{8}$	228.19	98
Ethanol	ADWIC	$\:{\text{C}}_{2}{\text{H}}_{5}\text{O}\text{H}$	46.07	99
Copper chloride	Sigma-Aldrich	$\:\text{C}\text{u}{\text{C}\text{l}}_{2}$	134.45	99

### Synthesis of graphene oxide (GO)

GO was prepared by oxidizing graphite with what is known as Hammer‘s improved method^108,109^. Briefly, 3 g of graphite was dispersed in 400 mL of a 1:9 mixture of phosphoric and sulfuric acids. Potassium permanganate was added gradually while the temperature was monitored. After the addition, the temperature was maintained at 50–55 °C for 24 h. The product was washed frequently with hydrochloric acid, followed by distilled water to remove residual sulfate, phosphate, and chloride ions. The drying was carried out in an oven at a temperature of 60 °C for 12 h.

### Synthesis of poly meta-aminophenol/graphene oxide (PmAP/GO) hybrids

The synthesis was based on the polymerization of 3-aminophenol in the presence of GO dispersion. In detail, the GO dispersion of 0, 3.3, 6.6, and 9.9 mg/mL were obtained using ultrasonic waves. In 100 mL of the aforementioned aqueous dispersions, 27.5 mmol of 3-aminophenol was added to the suspension. Sodium acetate was used in a small proportion (150 mg) to raise the initial polymerization pH value. The mixture was stirred for 30 min in an ice bath. The oxidizing agent, solution of APS, was added to the reaction at a fixed flow rate of 1.6 mL/min. The APS-to-monomer ratio value was set at 1.5:1. The reaction continued overnight at a temperature below 10 °C. The product was separated by vacuum filtration and washed on the filter paper with hydrochloric acid, followed by a large amount of ethanol. The product was left to dry inside the oven at a temperature not exceeding 60 °C. The resulting hybrids were labeled PmAP/GO_(x)_, where x is the initial GO concentration in the polymerization medium (mg/mL).

### Amino functionalized poly meta-aminophenol/APTES/graphene oxide (PmAP/APTES/GO)

**Scheme 1:**
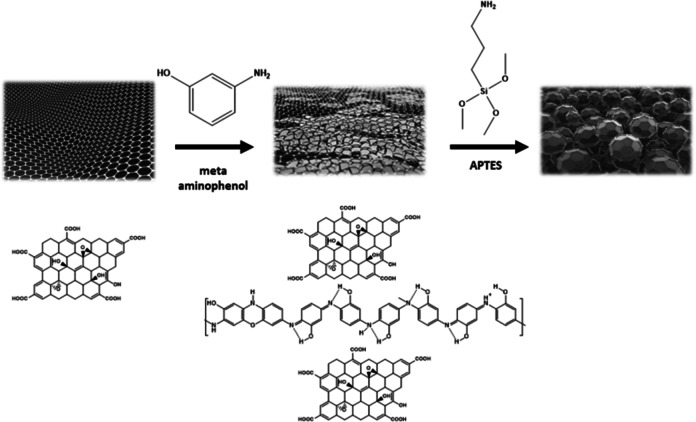
Illustration of the synthesis of PmAP/APTES/GO composite.

APTES was used for grafting the PmAP/GO_(x)_ composite. The condensation reaction between the ethoxy groups of APTES was carried out with the dense hydroxyl groups spread on the surface of PmAP/GO_(x)_ hybrids. The prepared PmAP/GO_(x)_ (1 g) was dispersed in a100 mL of water through ultrasonic waves. The temperature was raised to 70 °C before adding APTES (2 mL). The reaction continues for 3 h with reflux. The reaction was terminated by cooling. The precipitate was separated by filtration, followed by washing with distilled water to get rid of the unreacted residues of APTES. The precipitate was dried overnight at 60 °C. Scheme 1 shows an illustration of the step-by-step synthesis of (PmAP/APTES/GO_(x)_) nanohybrid.

### Characterization

The prepared PmAP/APTES/GO_6.6_ composite was characterized for its structure and morphology using several techniques. Fourier transform infrared spectroscopy (FT-IR) was recorded using a JASCO FT-IR-4100 spectrophotometer (Japan) within the range (4000–400 cm^-1^) using the potassium bromide disc technique. Powdered X-ray diffraction (PXRD) was performed on a GNR, APD 2000 PRO (Italy) with Cu Kα radiation (λ = 1.5418 Å). The sample was subjected to the range of 2θ = 5° to 70° with a scan rate of 8 (deg/min) at room temperature. Nitrogen adsorption-desorption measurements at 77.35 K were carried out using NOVA-touch (Quantachrome instrument, USA) to determine the pore structure of the prepared composites. Before the measurements, the sample was degassed at 100 °C for 2 h. The surface area was determined using the Brunauer–Emmett–Teller (BET) equation. The shape and microstructure were examined by scanning electron microscopy, SEM (FEI Quanta environmental, Spain). Transmission electron microscopy, TEM (JEM-2100 F, USA) is a potent method for observing local structures. Thermogravimetric analysis (TGA) was recorded on Shimadzu TG-50 under nitrogen of 10 mL min^-1^. The samples (5–10 mg) were heated from room temperature to 800 °C at a linear heating rate of 10 °C min^-1^. The value of the surface zeta potential was recorded on a Zeta potential analyzer (NICOMP, 380 ZLF, USA). UV-vis spectrophotometer (SPECORD 210 PLUS, Analytic Jena, Germany) was used to track down changing the adsorbed amount of Cu(II) on the composites. A bench pH meter (AD1030, Adwa, Hungary) was used to adjust the pH of the medium. X-ray Electron Spectroscopy (XPS) (Thermo Fisher Scientific, USA) was used to measure the spectra of C 1s, N 1s, and O 1s before and after Cu(II) uptake to understand the adsorption mechanism.

### Batch adsorption experiments

The adsorption performance was followed up by soaking the heterogeneous surface in a solution of Cu(II) ions. The baseline experiment involves soaking 20 mg of the composite in a 20 mL CuCl_2_ solution with a 300 mg/L concentration of Cu(II) at pH = 7, and 25 °C. Samples were periodically withdrawn through a medical syringe before filtration through a 0.45 μm polytetrafluoroethylene (PTFE) membrane. The filtrate was analyzed for the metal ion concentration while the exhausted composite was recovered for the recycling experiment. All experimental data were calculated as an average of triplicate measurements, with less than 3% relative errors. The following equations were used to get the adsorption $\:\text{c}\text{a}\text{p}\text{a}\text{c}\text{i}\text{t}\text{y}\:\text{a}\text{t}\:\text{a}\text{n}\text{y}\:\text{t}\text{i}\text{m}\text{e}\:({\text{q}}_{\text{t}},\:\text{m}\text{g}/\text{g})$ and removal efficiency:

3 $$\:{\text{q}}_{\text{t}}=\frac{\left({\text{C}}_{\text{o}}-{\text{C}}_{\text{t}}\right)\text{V}}{\text{m}}\:\:$$

4 $$\:\text{R}\text{e}\text{m}\text{o}\text{v}\text{a}\text{l}\:\text{E}\text{f}\text{f}\text{i}\text{c}\text{i}\text{e}\text{n}\text{c}\text{y}\:\left(\text{\%}\right)=\:\frac{{\text{C}}_{\text{o}}-{\text{C}}_{\text{t}}\:}{{\text{C}}_{\text{o}}}\times\:100$$

Where V is the volume of the solution (L); m is the weight of adsorbent (g); C_o_ and C_t_ are the concentrations of Cu(II) at a time equal to 0 and t, respectively.

The adsorption variables including the loaded amount of GO, pH, Cu(II) initial concentrations, adsorbent dose, and contact time were studied by changing one of these factors while keeping the rest of the others constant. Also, the reusability of the adsorbent was evaluated.

### Adsorption isotherms and kinetics

Several isotherm models such as linear and nonlinear Langmuir, Freundlich and D–R were applied to the adsorption experimental data to study the interaction of PmAP/ APTES/GO_6.6_ with Cu(II). Moreover, Linear and non-linear Pseudo-first-order (PFO) and pseudo-second-order (PSO) kinetic models were used to calculate the Cu(II) ions removal rate by the selected optimized composite. The equations of these models were given in the supporting information.

## Electronic supplementary material

Below is the link to the electronic supplementary material.

Supplementary Material 1

## Data Availability

All the data and materials are available in the manuscript and supporting information.
