# Supplementary material for: Fabrication of a poly(m‑aminophenol)/3-aminopropyl triethoxysilane/graphene oxide ternary nanocomposite for removal of Cu(II) from aqueous solution
Source: Sci Rep. 2025 Jan 27;15:3357. doi: 10.1038/s41598-025-85649-0 (PMC11772681; doi:10.1038/s41598-025-85649-0)
Supplement: Supplementary file 1 — Supplementary Material 1 [file 41598_2025_85649_MOESM1_ESM.docx]

**Fabrication of a poly(*m*‑aminophenol)/3-aminopropyl triethoxysilane/graphene oxide ternary nanocomposite for removal of Cu(II) from aqueous solution**

**Isotherm models**

The linear and nonlinear forms of the Freundlich model are given by the following equations ^1^.

$\ln q_{e}= \frac{1}{n}\ln C_{e}+\ln K_{F} (1)$

$$q_{e}=K_{F}C_{e}^{\frac{1}{n}} (2)$$

Equations (3) and (4), respectively, represent the linear and nonlinear forms of the Langmuir isotherm ^2^

$$\frac{C_{e}}{q_{e}}=\frac{C_{e}}{q_{\max}} + \frac{1}{q_{\max}K_{L}} (3)$$

$$q_{e}= q_{max} \left( \frac{K_{L}C_{e}}{1+ K_{L}C_{e}} \right) (4)$$

The separation factor ($R_{L}$) is one of the main parameters derived from Langmuir. It was first proposed by Weber and Chakravorti (1974) as a dimensionless constant denoted by Equation (5) ^3^. $R_{L}$ determines whether the shape of the isotherms is unfavorable ($R_{L}>1$), linear ($R_{L}$=1), favorable ($0<R_{L}<1$), or irreversible ($R_{L}$=0).

| $R_{L}=\frac{1}{1+k_{L}.C_{o}}$ | (5) |
| --- | --- |

The linear and nonlinear formulas of the Dubinin-Radushkevich (D-R) model are represented by Equations (6) and (‎7), respectively ^4^.

$$\mathrm{Ln}q_{e}= \mathrm{Lnq}_{m}-\beta\varepsilon^{2} (6)$$

$q_{e}=q_{s}e^{-\beta\varepsilon^{2}}$ (7)

Where $C_{e}$ and $q_{e}$ are the equilibrium Cu(II) ion concentration and adsorption capacity, $q_{\max}$ is the maximum adsorption capacity and $K_{F}$ is Freundlich constant which is related to the adsorption capacity. 1/𝑛 is adsorption intensity; it might indicate the relative distribution of the energy and the heterogeneity of the adsorbent sites. As ($0<\frac{1}{n}<1$) the adsorption is favorable. $K_{L}$ is the Langmuir constant. q_m_ is the monolayer capacity (mg g^-1^), and ε is the Polanyi’s potential. The value of ε can be written as: ε = RT ln[1 + 1/C_e_]. The value of β gained from the slope of D-R’s plot is utilized to calculate the mean adsorption energy (E, kJ mol^-1^) which was obtained from the following equation:

$$E=1/\left( -2\beta\right)^{0.5} (8)$$

**Kinetic models**

To inspect the mechanism of adsorption, two kinetic models were used to analyze the adsorption data; namely, linear and non-linear forms of pseudo-first-order (Eqs. 9 and 10, respectively) ^5^ and linear and non-linear forms pseudo-second order (Eqs. 11 and 12, respectively) ^6^.

$\ln\left( q_{e}-q_{t} \right)=\ln q_{e}-k_{1}t$ (9)

$q_{t}=q_{e}(1-e^{-k_{1}t})$ (10)

$\frac{t}{q_{t}}=\frac{1}{k_{2}q_{e}^{2}}+\frac{t}{q_{e}}$ (11)

$$q_{t}=\frac{k_{2}q_{e}^{2}t}{1+{k_{2}q}_{e}t} (12)$$

Where, q_t_ (mg/g) is the adsorbed amount of Cu(II) at contact time t. k_1_ (min^-1^), and k_2_ (g/mg min), are the rate constant of pseudo-first-order and pseudo-second-order, respectively

Moreover, the kinetic data were introduced into intraparticle diffusion model which is expressed by equation (13) ^7^:

$$q_{t}=k_{p}t^{1/2}+C \left( 13 \right)$$

*k_p_* is the rate constant of the intraparticle diffusion (mg /g min^0.5^) and *C* is a constant (mg /g) presenting data about the boundary thickness of the layer. The bigger *C*-value, the more influence of the boundary layer.

**Figure S1.** TGA-DTA curve for GO.

**Figure S2.** TGA-DTA curve for $\mathrm{PmAP}$.

**Figure S3.** TGA-DTA curve for ${PmAP/GO}_{(6.6)}$.

**Figure S4.** Linear and nonlinear regression for the adsorption data of Cu(II) ions onto $PmAP/APTES{/GO}_{(6.6)}$. [Cu(II)]_o_=150 mg/L, adsorbent dosage 0.01 g, temperature 25 °C, stirring speed 120 rpm, contact time 4 h (a) PFO model; (b) PSO model.

**Figure S5.** Effect of Cu(II) initial concentration; adsorbent dosage 0.01 g, temperature 25 °C, stirring speed 120 rpm, contact time 3 h., and pH 7.

**References**

1 Freundlich, H. Over the adsorption in solution. *J. Phys. chem* **57**, 1100-1107 (1906).

2 Langmuir, I. The constitution and fundamental properties of solids and liquids. Part I. Solids. *Journal of the American chemical society* **38**, 2221-2295 (1916).

3 Weber, T. W. & Chakravorti, R. K. Pore and solid diffusion models for fixed‐bed adsorbers. *AIChE Journal* **20**, 228-238 (1974).

4 Dubinin, M. The potential theory of adsorption of gases and vapors for adsorbents with energetically nonuniform surfaces. *Chemical reviews* **60**, 235-241 (1960).

5 Lagergren, S. K. About the theory of so-called adsorption of soluble substances. *Sven. Vetenskapsakad. Handingarl* **24**, 1-39 (1898).

6 Elsherbiny, A. S., Rady, A., Abdelhameed, R. M. & Gemeay, A. H. Efficiency and selectivity of cost-effective Zn-MOF for dye removal, kinetic and thermodynamic approach. *Environmental Science and Pollution Research*, 1-16 (2023).

7 Weber Jr, W. J. & Morris, J. C. Closure to “kinetics of adsorption on carbon from solution”. *Journal of the Sanitary Engineering Division* **89**, 53-55 (1963).
